# Mechanosensitive control of plant growth: bearing the load, sensing, transducing, and responding

**DOI:** 10.3389/fpls.2015.00052

**Published:** 2015-02-23

**Authors:** Bruno Moulia, Catherine Coutand, Jean-Louis Julien

**Affiliations:** ^1^NRA, UMR 547 PIAFClermont-Ferrand, France; ^2^Clermont Université, Université Blaise Pascal, UMR 547 PIAFClermont-Ferrand, France

**Keywords:** mechanobiology, biomechanics, thigmomorphogenesis, wind, turgor pressure, curvature, mechanotransduction, stress

## Abstract

As land plants grow and develop, they encounter complex mechanical challenges, especially from winds and turgor pressure. Mechanosensitive control over growth and morphogenesis is an adaptive trait, reducing the risks of breakage or explosion. This control has been mostly studied through experiments with artificial mechanical loads, often focusing on cellular or molecular mechanotransduction pathway. However, some important aspects of mechanosensing are often neglected. (i) What are the mechanical characteristics of different loads and how are loads distributed within different organs? (ii) What is the relevant mechanical stimulus in the cell? Is it stress, strain, or energy? (iii) How do mechanosensing cells signal to meristematic cells? Without answers to these questions we cannot make progress analyzing the mechanobiological effects of plant size, plant shape, tissue distribution and stiffness, or the magnitude of stimuli. This situation is rapidly changing however, as systems mechanobiology is being developed, using specific biomechanical and/or mechanobiological models. These models are instrumental in comparing loads and responses between experiments and make it possible to quantitatively test biological hypotheses describing the mechanotransduction networks. This review is designed for a general plant science audience and aims to help biologists master the models they need for mechanobiological studies. Analysis and modeling is broken down into four steps looking at how the structure bears the load, how the distributed load is sensed, how the mechanical signal is transduced, and then how the plant responds through growth. Throughout, two examples of adaptive responses are used to illustrate this approach: the thigmorphogenetic syndrome of plant shoots bending and the mechanosensitive control of shoot apical meristem (SAM) morphogenesis. Overall this should provide a generic understanding of systems mechanobiology at work.

## Introduction

Land plants continuously encounter mechanical challenges from without and within. External mechanical loads are imposed by the wind, rain, neighboring plants or solid substrates. The external bending loads imposed by winds induce a syndrome of mechanosensitive growth responses in the aerial stems of plants known as thigmomorphogenesis. The activity of their meristems is modulated to stunt vertical growth and stimulate an increase in girth, thereby making the plant more wind-resistant (see Telewski, 2006; Coutand, 2010; Monshausen and Haswell, 2013 for reviews). Internal loads may be imposed by the plant's own weight, inertial forces and the large hydrostatic turgor pressure in cells. Even meristems, although protected from many external mechanical loads by young leaves in the shoot apical bud or by the bark in the lateral cambium, are under considerable direct mechanical stress due to the inner turgor pressure and the mechanical barriers imposed by neighboring organs or tissues (e.g., Couturier et al., 2012; Baskin and Jensen, 2013). Therefore, precise mechanosensitive control of the magnitude and direction of growth is required so that the size, shape, and edges of the growing organs and tissues are produced in a regular and stable way (Hamant, 2013). It follows that acclimation responses of growth and morphogenesis have been naturally selected to reduce the risk of breakage or explosion of plant parts during growth and development.

These two adaptive responses, stem thigmomorphogenesis and meristem growth, ultimately rely on mechanosensing of the internal mechanical state of the living cells of the plant as a cue for the regulation of growth and morphogenesis (Coutand, 2010; Moulia et al., 2011; Hamant, 2013; Monshausen and Haswell, 2013). Mechanosensing occurs at the cell level, yet mechanical stimulation involves loads that act on the whole organ, either at its boundaries (e.g., for wind-drag) or across its full volume (e.g., weight, inertial forces or turgor pressure). Therefore, changes in the mechanical state of tissues and cells that trigger cell mechanosensing depend on the load, on the mechanical structure of the organ, and on the mechanosensitive structure. The mechanosensitive structure is defined as the location and amount of mechanosensitive tissues involved in a response. Some mechanosensed modulations of growth and morphogenesis are triggered through long-distance internal signaling so the connection between the mechanosensitive structure and the responding structure also needs to be borne in mind (Coutand, 2010; Moulia et al., 2011).

Analyzing and modeling the biology of mechanosensing and of mechanosensitive growth responses thus involves three phases (Moulia et al., 2011). (i) Biomechanical analysis reveals how mechanical loads are distributed over the constitutive plant tissues and cells. (ii) The local mechanosensitive pathways are analyzed in the sensing cells. (iii) Mechanobiological integration combines the models describing how the plant's local mechanosensing relates to global growth responses. Other comprehensive reviews have focused on the local analysis of the mechanosensitive pathway or on the global responses of growth and morphogenesis (e.g., Braam, 2005; Telewski, 2006; Coutand, 2010; Monshausen and Haswell, 2013 to cite just a few). Our purpose instead is to review the integrative aspects, tracing them down the scale from the effect of the load on the plant to the effects on tissue elements and cells, and then up the scale from mechanosensitive gene expression to the growth and morphogenetic responses of the organ. Two mechanosensitive growth responses have been particularly extensively studied in the last two decades: thigmomorphogenesis of stems responding to external bending loads, and growth and morphogenesis of the shoot apical meristem (SAM). In particular, we aim to illustrate how integrative models combining structural mechanics with mechanosensory biology have been instrumental in understanding how mechanical loads are distributed within the plant, defining the heterogeneous stress and strain fields. We explain how the models become key experimental tools to qualitatively and quantitatively assess hypotheses about sensory mechanisms (e.g., does sensing occur through stretch-activated channels or wall-associated transmembrane proteins? Is strain sensed or is stress sensed or both?) or the influence of organ geometry and tissue distribution on the magnitude of mechanosensitive responses. This review is designed for a general biologist audience and aims to help biologists master the mechanical models they need for mechanobiological studies. There is no need for an advanced background in mechanics, mathematics or modeling as the crucial equations are introduced both verbally and graphically. The list of the models and of their acronyms can be found in Table 1.

**Table 1:** **List of models with their acronyms and references**.

**Acronym of the model**	**Full name**	**References**
CBmS	Composite beam model of the Stem (in flexion)	Moulia and Fournier, 1997; Gibson et al., 1988; Coutand and Moulia, 2000
PVm	Pressurized vessel model of the SAM	Hamant et al., 2008; Traas and Hamant, 2009
FEm	Finite elements model of two patches of the L1 + L2 tissues	Hamant et al., 2008
2D SFm	Two-dimensional cellular stress feedback model	Hamant et al., 2008
S^3^m	Sum of strain-sensing model	Coutand and Moulia, 2000; Coutand et al., 2009; Moulia et al., 2011
SAM SFm	Integrative SAM stress feedback model ()	Hamant et al., 2008
GSFm	Growth-strain feedback model	see replies to Hamant et al., 2008 by Schopfer and Meyerowitz in Science e-letters

## Mechanical characteristics of loads and their heterogeneous distribution within the plant

Some central concepts are introduced briefly here that are essential when taking a mechanical view of plant structure. More complete primers in plant mechanics, including lists of definitions, are available (Boudaoud, 2010; Moulia et al., 2011; Moulia, 2013).

### Crucial mechanical concepts

Mechanical loads may involve the action of localized forces (e.g., intermittent contact with a neighboring stone or organ) or distributed loads (e.g., external drag by wind flow, self-weight or the internal turgor pressure of the living cells). Some loads are static or quasi-static (their rate of change is slow), whereas others are dynamic so inertial forces due to the acceleration of mass need to be considered (e.g., wind-induced oscillations) (Rodriguez et al., 2008; Pivato et al., 2014).

Under the action of internal and/or external load(s) a solid body such as a plant organ can be globally displaced. This displacement involves a translation of the center of mass of the body and the body might rotate around the center of mass, described in terms of velocity. In addition, parts of the body may be displaced relative to one another, resulting in a change of shape, called deformation. These deformations are measured locally by strains, written as ε. Strains may be linear or shear (angular) and are measured in relative units (i.e., strains are dimensionless). Straining stretches bonds and causes slide/shear of internal elements, thereby allowing internal reaction forces to build up. In this way the mechanical load is distributed through the material with the storage of elastic strain energy across the deformed body until an internal and external mechanical equilibrium is achieved, i.e., all the forces and moments acting on the body are balanced. The density of the resulting internal forces, i.e., the internal forces per unit of area, is called stress, written as σ and measured in Pascal (Pa) which is equivalent to N.m^−2^. Strains and stresses can be very heterogeneous across the body. Deformation is characterized by the strain and stress fields, i.e., the amount of strain and stress at every location of the body at a given time. The strain and stress fields in a given load situation therefore, measure the mechanical state of a body like a cell, an organ or a plant.

The amount of stress produced by straining is linked to the rheological properties of the material. Rheology can be modeled in a so-called constitutive equation. If the stress increases linearly with increasing strain and linearly reverts during unloading, the material behaves as a linear elastic material. The slope of the stress-strain curve is called the Young's modulus, such that a stiff, rigid material has a high Young's modulus. Over a certain threshold, some materials may yield plastically (irreversibly). From this point, in pure plastic materials, stress does not increase further with strain, but in visco-plastic materials it varies depending on the strain rate. The growing cell wall has a visco-plastic rheology (called the Bingham flow model, see Dumais, 2013 for more details). Finally, when internal forces overcome the strength of the material, fracture occurs.

### Posing the problem: experimental setups

**Figure 1:**
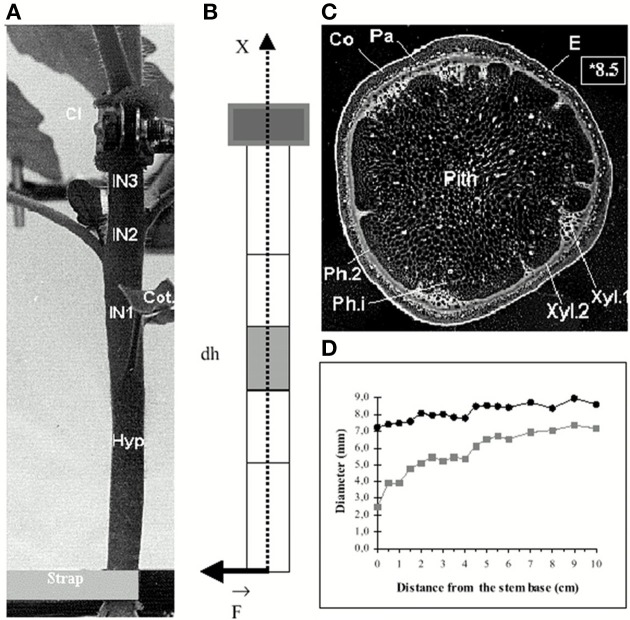
**Morphological and anatomical structure of a stem submitted to an external bending load from Coutand and Moulia (2000), Journal of Experimental Botany, by permission of the Society for Experimental Biology. (A)** Side view of the basal part of the stem base before the application of bending. The stem is grown in hydroponics, and clamped below the primary growth zone, so that bending does not affect its position (Cl, metal clamp; IN 1–3, internodes; Hyp, hypocotyl; Cot, cotyledons. **(B)** Idealized geometrical scheme **(A)** as a cantilever beam. **(C)** Negative photograph of a cross-section (note the quasi-circular shape and the concentric layers of tissues (E, epidermis; Co, collenchyma; Pa, parenchyma; Ph.2, metaphloem; Ca, cambium; Xyl.2, metaxylem; Xyl.1, protoxylem; Ph.i, internal phloem). **(D)** Changes in the external diameter (●) and of the diameter of the pith (■) along the basal part of the stem.

Thigmomorphogenetic experiments are generally conducted in the lab by subjecting single plant stems to static bending. Typically a stem is bent by moving the top of the stem laterally while the base is anchored immobile in the soil (Telewski and Pruyn, 1998). Alternatively, the stem is first clamped in a vertical position with the roots bathed in a liquid medium, then the basal part of the stem is displaced (Figure 1). The latter setup decouples the effects of stem bending from tilting the apical growth zone (Coutand et al., 2000). Dynamic loading can also be imposed by vibrating the plant, for example (Der Loughian et al., 2014).

**Figure 2:**
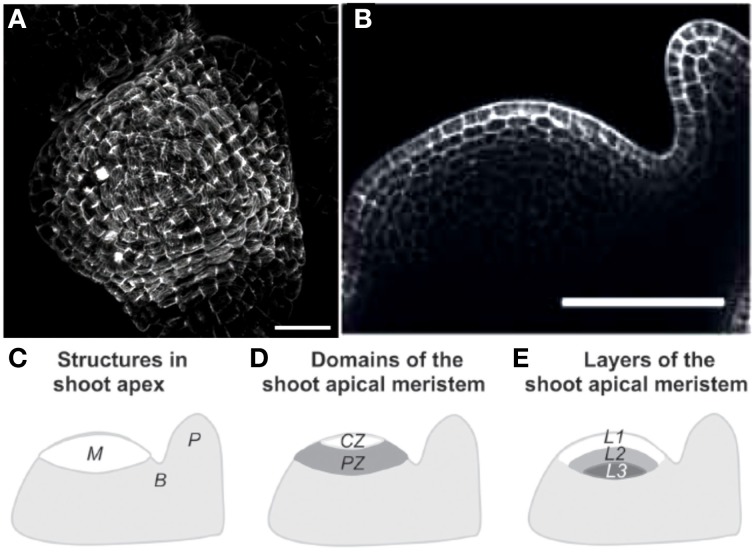
**Structure of the shoot apical meristem (SAM). (A)** View of an *Arabidopsis thaliana* SAM from above Hamant et al. (2008), reprinted with permission from AAAS. **(B)** Side view of a tomato SAM (Robinson et al., 2013). **(C–E)** Generalized schematic representations of a typical dome-shaped shoot apex bearing a cylindrical young primordium. **(C)** Major structures. M, shoot apical meristem; P, organ primordium; B, boundary between the meristem and the primordium. **(D)** Morphological domains of the SAM. CZ, central zone, PZ, peripheral zone where new organs are generated. **(E)** Internal organization of the SAM. L1, presumed epidermis, L1 and L2, tunica layers, L3, corpus from Robinson et al. (2013), Journal of Experimental Botany, by permission of the Society for Experimental Biology.

For most SAM experiments, the meristem is cut from the stem and the surrounding young leaves are removed (Figure 2). The isolated SAM is then cultured on a growth medium. Three types of mechanical perturbations have been used on excised SAM. The osmotic potential of the bathing solution can be changed to transiently manipulate the inner turgor pressure of the cells (Peaucelle et al., 2011). External loads, such as lateral compression of the whole meristem, can be applied mechanically (Hamant et al., 2008). Alternatively, outer cells can be ablated to create holes, thereby modifying the mechanical structure and state of the SAM (Hamant et al., 2008). To our knowledge, the mechanical states of intact SAM have not been studied so far.

In both experimental systems, the next step is to estimate the amount and spatial distribution of the changes in mechanical state (stress and strains). This can be done as (i) the external mechanical load and the mechanical structure of the organ are known, and (ii) the changes imposed by the experimenter or by the environmental conditions are measured. However, to estimate changes that occur at very different scales, we need to consider how a change in a unit of the cell wall or tissue affects the whole organ (and vice versa). For this, a mechanical model needs to be developed, using a scientific method originating from mechanical engineering called integrative structural mechanics (ISM) modeling. More complete coverage of ISM modeling can be found in Moulia et al. (2011).

### Integrative models in mechanics

**Figure 3:**
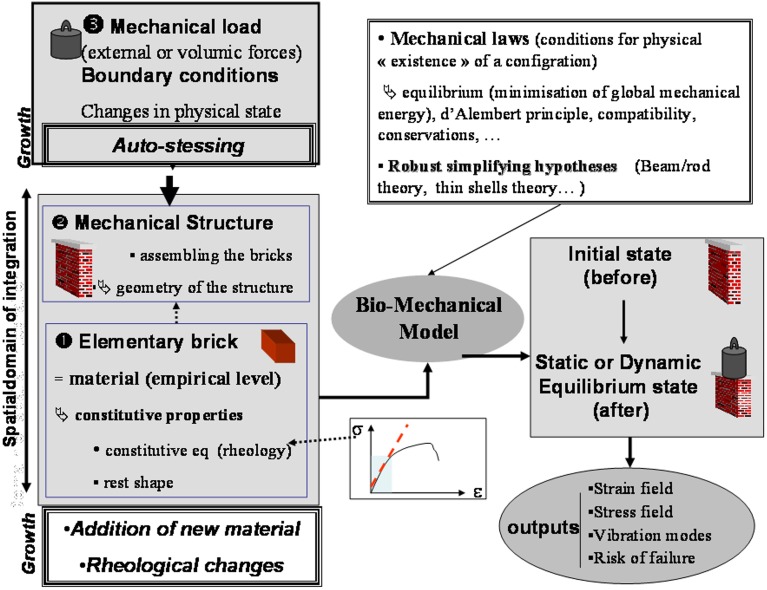
**Structure of an integrative structural mechanics (ISM) model from Moulia et al. (2011), by permission of Springer-Verlag Berlin Heidelberg.** The structure of an ISM model for use in plant biomechanics. ISM models consider (at least) two scales in the system: a scale of phenomenological empiricism called the material scale, and a scale of mechanistic spatial integration, the mechanical structure. The internal and boundary loads (inputs) result in a change in mechanical state that can be calculated using mechanical principles and robust simplifying theories. ISM models can produce various outputs characterizing mechanical state or dynamics, such as strain (ε) and stress (σ) fields, vibration modes, or rupture risk factors.

The general structure of an ISM model is shown in Figure 3, using schematic graphical conventions that will be used throughout this review. The major aspects of the models can be understood independently of the detailed model equations. First, the constitutive materials of the structure, the elementary “bricks” or units, are defined and the rheological properties of these elements specified. Do they behave elastic or do they undergo viscoplastic deformations? Are they isotropic, displaying the same properties in all directions? If they are anisotropic, in which direction is the anisotropy? (Coutand and Moulia, 2000; Hamant et al., 2008; Baskin and Jensen, 2013). It is very important to know the shape of these elements at rest (without a load) and whether the shape is dependent on other physical variables such as temperature, water status or time (e.g., Moulia, 2000; Hamant et al., 2008). The size of these elements is not prescribed as the mechanical structure of plants is multiscale and the scale at which to work is mostly a matter of informed choice (Boudaoud, 2010; Niklas and Spatz, 2012; Gibson, 2013). Next, the structure is defined by specifying how the elements are assembled (topology) and displayed geometrically. Note that to model heterogeneous organs, several materials may need to be considered (e.g., Moulia and Fournier, 1997; Coutand and Moulia, 2000; Routier-Kierzkowska et al., 2012). Finally, the mechanical loading applied to the structure is defined, as well as including any boundary conditions, which are displacement or force constraints at the boundaries of the structure.

With these three steps the model is now fully defined. When input values are known like the load applied or the structural change (e.g., making a hole in the structure), the state of the structure in the loaded state can be computed because mechanical laws specify(i) the conditions for equilibrium (static or dynamic), and (ii) the compatibility of strains between adjacent material elements or boundaries. Depending on the structure's geometry, some simplifying hypotheses can be used for calculations, e.g., beam or shell theories. In some cases the problem can even be solved analytically (e.g., Hamant et al., 2008). Mostly, however, numerical methods are required for computations. The outputs of such models can be multiple: knowledge of strain and stress fields, the velocity of the top of a plant, or bending rigidity, etc.

Plants are open systems. If cells grow or differentiate the amount and/or rheology of constitutive materials may change (e.g., cell wall maturation) and will need to be accounted for in a model. This has important implications in formulating the mechanical problems that are specific to biomechanical models (Moulia and Fournier, 2009). For example, for the tree gravitropic reaction, the problem can be solved by using beam theory hypotheses but requires an incremental formulation of the problem (e.g., Fournier et al., 1994; Fourcaud et al., 2008; Coutand et al., 2011) to take into account the growth and shrinkage of the cell wall rest-length during wood maturation (Coutand et al., 2011; Pot et al., 2014).

### Load distribution from the plant to mechanosensitive cells

Two examples of analyzing load distribution from the scale of the whole plant down to the scale of mechanosensitive cells will be presented.

### Composite beam model of the stem subjected to bending

The dicot stem is composed of several tissues of very variable stiffness, e.g., epidermis, parenchyma, sclerenchyma, and wood. Growth activity is concentrated in (i) the primary growth zone just below the SAM and (ii) the cambial zone, a thin shell of 1–20 layers of meristematic cells near the lateral surface of the stem, just beneath the bark. The primary growth zone is responsible for longitudinal growth, and the cambial zone for most radial expansion.

As the stem is generally a slender structure (the diameter:length ratio is less than 1/20) (see Figure 1) and its constitutive tissues are in transverse layers, the mechanical analysis can be simplified using the theory of heterogeneous composite beams or rods (Gibson et al., 1988; Moulia and Fournier, 1997), reframed in a mechanobiological context (Coutand and Moulia, 2000), and called the the Composite Beam model of the Stem (CBmS) in the following.

**Figure 4:**
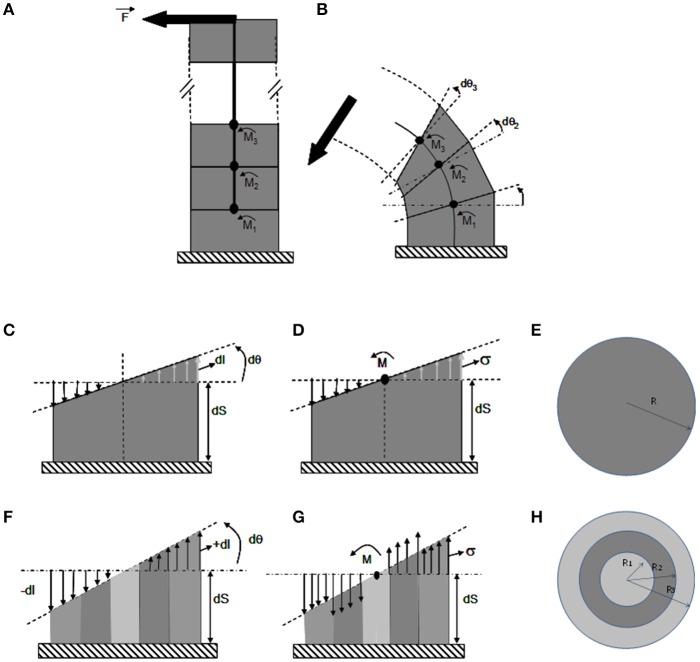
**The ISM beam model of pure bending of a stem.** ISM model used to analyze stem bending experiments, using the theory of composite heterogeneous beams in a cantilever setting. **(A)** Unloaded beam. The beam is composed of a pile of (virtual) slices of infinitesimal thickness delimited by (virtual) successive cross-sections, along a central line. **(B)** Loaded beam. Under bending moment $\overset{⌢}{M}$(ζ), the beam curves. Each cross-section rotates by a small angle dθ(ζ), with ζ being the position along the stem and x, y the coordinates within the current cross-section of the stem. **(C–E)** Detailed side **(C,D)** and top **(E)** views of a bent slice in a homogeneous stem. **(F–H)** Detailed side **(F,G)** and top **(H)** views of a bent slice in a heterogeneous stem made of one stiff (dark gray) and two compliant (light gray) concentric annuli of tissues. **(C,F)** Strain distribution across the cross-section. Note that the cross-section remains flat during the bending and only rotates respective to the previous cross-section at the bottom of the slice by an angle dθ, irrespective of the anatomy of the stem. The spatial rate of change in angle of the successive cross-sections is the stem curvature $C=\frac{d\theta }{dS}$. Accordingly, the stem is elongated on the convex side by *dl*(*x*, *y*) > 0 and shortened on the concave side by *dl*_(*x*,*y*)_ < 0, with no change on the central (neutral) line. The longitudinal strain $\varepsilon_{LL}=\frac{dl}{dS}$ is thus maximal at the periphery on the sides of the slice that face downwards and away from the orientation of the bending force. The heterogeneous anatomy of the stem has no effect on the relative distribution of strain across the cross-section, which remains linear and is given by ε_*LL*,*y*_ = *y* · (*C* − *C*_0_). Straining allows for internal reaction forces, which density is measured by stresses, to build up balancing the effect of the external load. Therefore, the amount of change in stem curvature (and hence the global amount of straining) only depends on the amount of external bending moment and on the overall bending stiffness of the stem. **(E,F)** Stress distribution in the cross-section. For elastic constituents, the stress is equal to the strain multiplied by the Young's modulus: σ_*LL,x,y,i*_ = ε_*LL,x,y*_*E*_*LL,i*_ where *E*_*LL,i*_ is the longitudinal elastic modulus of material i. In homogeneous stems stresses parallel strains. However, on a stem with a heterogeneous anatomy **(F)** the stresses also depend on the local stiffness of the tissue and they de-correlated with strains across the cross-section (with maximal stresses possibly occurring inside the stem).

This mechanical modeling is detailed step by step in Figure 4. Only longitudinal strains and stresses will be considered, noted by the subscript LL, as transverse shearing can be neglected when analyzing slender structures, which exhibit pure bending. The material element in the CBmS is a small volume of tissue. Two types of tissues, broadly organized into three concentric rings, were defined. Tissues such as parenchyma or phloem were treated as compliant materials, and tissues like wood as stiff materials. These elements are assumed to behave in the linear elastic range as has been confirmed experimentally (Coutand et al., 2000). These tissue elements are then assembled into infinitesimal slices of dS thickness according to the known anatomy of the stems. Finally, the stem can be viewed as a pile of infinitesimal slices, glued one next to another along an imaginary line inside the stem, called the neutral line.

During bending experiments one end of the stem is fixed and one end is free to move. The stem therefore, operates mechanically as a cantilever subjected to a local force $\vec{F}$ transverse to the stem. The action of $\vec{F}$ depends on the amount of the force F and on the lever arm L, i.e., the distance from the application point of the force to a given slice. This mechanical amplification effect can be modeled using a quantity called the bending moment $\overset{⌢}{M}$, the magnitude of which is M = F.L (Equation 1).

A central property of beam bending is that each cross section remains flat and orthogonal to the neutral line all along the deformed beam. A change in $\overset{⌢}{M}$ will thus induce a relative rotation (through an angle *d*θ) of two successive stem slice cross-sections. The effect of the rotation is an increment of length *dl* on the tensed side and a decrement of *dl* on the compressed side. The ratio $C=\frac{d\theta }{dS}$ (Equation 2) is called the curvature. It measures the spatial density (rate) of bending rotation and $\varepsilon_{LL}=\frac{dl}{dS}$ (Equation 3) measures the longitudinal strain. The strain at any point located at a distance *y* from the cross-section center can be computed as the product of the change in curvature and the distance *y* to the central line of the stem, ε_*LL,y*_ = *y* · (*C* − *C*_0_) (Equation 4), where *C*_0_ is the initial stem curvature before the load (if the initial configuration of the stem is straight). The value of the longitudinal strain thus varies with the position along the beam, being maximal at the beam periphery (*y* = *R*) along a radius aligned with $\vec{F}$.

Straining allows internal reaction forces to build up to balance the effect of the external load. For elastic constitutive materials, the stress is calculated as the strain multiplied by the appropriate Young's modulus: σ_*LL,x,y,i*_ = ε_*LL,x,y*_*E*_*LL,i*_ (Equation 5) where *E*_*LL,i*_ is the longitudinal elastic modulus of the i^th^ material and x, y are the spatial coordinates within the cross section.

The amount and distribution of stresses and strains can then be calculated so that they balance out $\overset{⌢}{M}$ (as detailed in supplemental data). This yields ε_*LL*_ = *y*(*C* − *C*_0_) = *My*/(*E*_*soft*_*I*_1_ + *E*_*stiff*_*I*_2_ + *E*_*soft*_*I*_3_) and σ_*LL*_ = *E*_*i*_ε_*LL*_, (Equation 6). *E*_*i*_ is the Young's modulus of the *i*th tissue slice. *I*_*j*_ measures the effect of all the internal lever arms of the resisting stresses in a given tissue and works out as $I_{i}=\pi \frac{R_{Out,i}^{4}-R_{in,i}^{4}}{4}$ where *R*_*out,i*_ and *R*_*in,i*_ are the outer and inner radii of the *j*th annulus of tissue. These formula specify the consequences of loading (F), stem geometry and anatomy (L, I_j_), and material stiffness (E_i_) on the stress and strain fields. Note that if strains increase linearly from the center to the periphery, stress may be distributed non-continuously. Another striking property of beam bending apparent in the previous equation is that changes in cross-sectional geometry have much more effect on stresses and strains than changes in material stiffness. For example, for a given load F, doubling the elastic stiffness of all the tissues halves the longitudinal strain without changing longitudinal stress. Doubling the stem radius however (keeping the same proportion of concentric tissues) reduces both strains and stresses at the stem periphery 8-fold. This is another example of mechanical amplification by lever arms. We will see that this has profound consequences on the mechanical stability and the mechanosensitivity of a given stem. Finally, stresses and strains are highly anisotropic, with their principal component lying longitudinally along the length of the stem.

#### Shell model of the SAM under internal pressure load

The SAM, a group of continuously growing and dividing cells, is a dome-shaped structure (Figure 2) composed of two outer layers, named L1 (the outer epidermis) and L2, and an inner bulk of cells named L3. Future definite lateral organs, like leaves, sepals, or petals, are initiated as primordia, bulges at the periphery (see reviews by Kwiatkowska, 2008; Burian et al., 2013; Robinson et al., 2013). Between the primordium and the apical dome, a saddle-shaped boundary forms which later becomes a sharp crease that separates the growing primordium from the SAM. The thin-walled fully turgid cells in the SAM are under considerable mechanical load from turgor pressure and cell-to-cell mechanical interactions known as “tissue tensions.”

**Figure 5:**
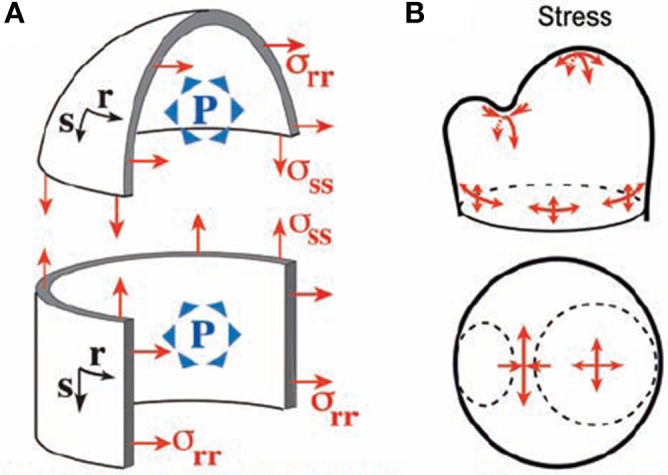
**The thin-walled pressurized vessel model of the shoot apical meristem.** Integrative structural mechanics (ISM) model used to analyze the loading of the SAM by internal turgor pressure, using very thin shell theory (Hamant et al., 2008), reprinted with permission from AAAS. **(A)** The SAM modeled as a pressurized vessel. Each point has a coordinate in the orthoradial (*r*) and meridional (*s*) direction and P is pressure. **(B)** At the top of the apical dome, represented as a spherical dome, the stress is isotropic. If the flanks of the meristem are represented as a cylinder, the stress is greater in the circumferential (orthoradial) direction than along the meridian and strongly anisotropic stresses occurrs on the flanks of the meristem. Maximal stress anisotropy occurs at the saddle-shaped boundary between the primordium and the central dome.

The mechanical analysis of the meristematic dome has been performed by Hamant et al. (2008) and Traas and Hamant (2009), first giving rise to the Pressurized Vessel model (PVm). The meristematic zone is modeled as a vessel according to thin-shell theory. The vessel “wall” corresponds to the outer wall of the L1 layer, which is thicker than the other walls and likely to bear much of the load due to the turgor pressure of inner cells. The modeled vessel wall is built of thin shell elements of infinitesimal dimensions ds and dr and of thickness t. Their material properties are considered to be homogeneous across the SAM (for discussion see Baluska et al., 2003). Hamant et al. (2008) proposed that the elements should be linear elastic, but this is not necessary as the material could equally well be viscoelastic. The only restriction is that the material element should not show pure plastic properties as this would induce loss of rheological homogeneity during loading. These shell elements are smoothly assembled (i.e., essentially they are virtually “glued” together along their sides) into a typical SAM structure, modeled geometrically by combining three simple adjoining structures (Figure 5). (i) The apical dome is represented as a spherical dome of radius R. (ii) The flanks of the meristem are represented by a cylinder of radius R. (iii) Where relevant an incipient primordium is represented by a smaller lateral dome. Each point of the vessel “wall” is characterized by its coordinates in orthoradial (*r*) and meridional (s) directions (Figure 5A). The difference between a beam slice and a shell element is that each shell element can display curvatures in two directions (i.e., C_*rr*_ along an orthoradial line, C_*ss*_ along a meridian) and may also display a twist (C_*rs*_). Note that in the central or primordial domes, C_*ss*_ and C_*rr*_ have the same sign, the concave surface faces into the meristem and C_*rs*_ = 0), whereas in the boundary, C_*ss*_ and C_*rr*_ have opposite signs. Importantly, this geometry is assumed to be under static equilibrium, so that the model only aims at calculating the stresses required to achieve this static equilibrium in the specified geometrical configuration and strains are unknown. The load is considered to be a uniform and constant inner pressure P. The effect of this internal load in the model can be described verbally as follows. Each shell element builds up stresses in three directions, and its stress state is thus represented by a stress tensor (σ_*ss*_, σ_*rr*_, σ_*rs*_), with σ_*ss*_ and σ_*rr*_ being tensions in the meridional and orthoradial directions, and σ_*rs*_ is a shear stress within the vessel wall (a kind of internal friction stress). The values of each stress component can be fully estimated using the conditions of static equilibrium, and depends on the curvatures in each zone of the SAM. This was solved analytically at specific points of local symmetry.

In the central dome, equilibrium yields $\sigma_{ss}=\sigma_{rr}=\frac{PR}{2}$, σ_*rs*_ = 0 (Equation 7). The tensile stresses are isotropic as they have same value in the *s* and *r* directions. In the flanks of the meristem cylinder, σ_*rr*_ = *PR*, $\sigma_{ss}=\frac{PR}{2}$ (Equation 8), so the stress is highly anisotropic, being twice as high in the circumferential direction as in the longitudinal direction.

In the saddle-shaped boundary between the apex and a primordium, one may assume that the orthoradial curvature is approximately the curvature of the dome $Crr\approx \frac{1}{R}$ whereas the meridional curvature C_*ss*_ is much higher (in absolute terms). The stresses matching static equilibrium are therefore: $\sigma_{ss}\approx \frac{P}{2C_{ss}},\sigma_{rr}\approx \frac{P}{2C_{rr}}\approx \frac{PR}{2},\left|\sigma_{rr}\right|≻≻\left|\sigma_{ss}\right|$ (Equation 9). The outer wall of the SAM is under tension across the crease, but in compression along the crease. The amount of the stress depends on P and on one of the two curvatures C_*rr*_ and C_*ss*_, with higher curvature inducing lower stresses for a given P. The absolute amount of stress is much higher across the crease, and the stress distribution is highly anisotropic.

**Figure 6:**
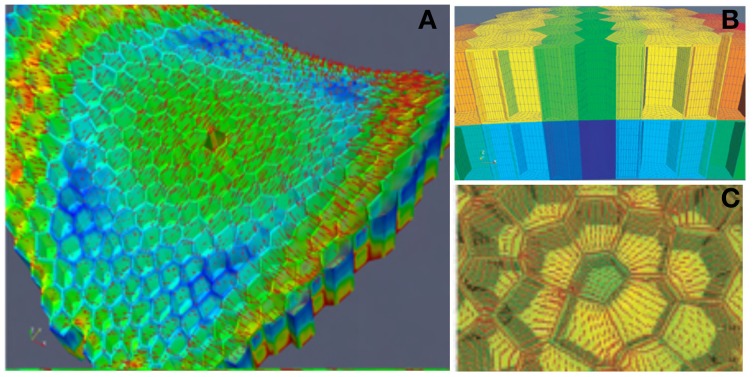
**Finite elements model (FEm) of a patch of the L1 + L2 layer of the SAM.** ISM model for the numerical mechanical analysis of a small patch of the SAM with full cellular resolution. The example here displays the model (and the numerical simulation of its stress-field output) of a patch at the boundary between the primordium and the central dome, with the ablation of one L1 cell from Hamant et al. (2008), reprinted with permission from AAAS. **(A)** General view of the FEm of the patch in the primordium boundary zone from above indicating the simulated pattern of principal stress directions (red lines) on the outer surface of meristem tissue. Colors indicate relative values of stress (blue, low; green, medium; red, high). **(B)** Side view of the outermost cell layers L1 and L2. **(C)** Detail of the stress pattern around one hole due to cell ablation.

This model of the SAM as a thin-walled pressurized vessel was very instructive. However, it did not provide detailed insights into how stress is distributed in the cell walls of a given SAM zone. It was therefore, complemented by a second “zoom-in” model at the scale of a small patch of tissue in the L1 and L2 layers (Figure 6). This model is a Finite Elements model (FEm) of two patches of the L1 + L2 tissues, one in the primordium-boundary zone and one at the top of the dome. Detailed specification of the geometry of the cell walls of this patch was achieved by experimental measurements. Elements were meshed piecewise to form plates of finite size. Just as for the PVm, the material was assumed to be homogeneous, but also linear elastic (with no plastic deformation or growth) and the load resulted from a uniform internal pressure putting the L1 layer into a curved configuration. No internal pressure within the cells of the L1 and L2 layers was considered as the effect of uniform pressure among the neighboring cells cancels out within a cell layer. The boundaries of the patch were given the proper saddle shape of the primordial boundary or the hemispherical shape of the dome. The mechanical equilibrium state was then computed numerically. This was done both for the intact patch, and for a patch in which one or two cells were ablated (i.e., their outer walls were deleted from the model). Making one hole in the patch redirects the orientation of the main stresses to surround the hole, and increases the magnitude of these stresses. When holes are made in two adjacent elements, highly anisotropic stresses are induced between the two holes, even if they are positioned at the tip of the dome where stresses are normally isotropic. Note that as the elastic rheology of the cell wall was specified, the FEm could be used to estimate not only the stress distribution within the cell walls, but also the elastic strains of the walls (this was not possible using the PVm).

## Mechanosensing and mechanotransduction

Now that we have tracked the distribution of stresses and strains within the two types of organs and the two types of loads, we can study how the cells sense their local mechanical state. Models can be helpful tools at this stage too to define quantitative behaviors and to deduce which variable is sensed.

### Strain-sensing or stress-sensing? does it matter?

Mechanobiologists have paid relatively little attention to the issue of whether plant cells sense stress or strain, implicitly assuming that mechanical “stress” is the variable of interest perhaps due to semantic confusion with physiological “stress” (Moulia et al., 2011). However, it is important to remember that stresses and strains do not parallel in heterogeneous constitutive materials (e.g., stems) or in materials behaving in the plastic range (e.g., during growth). Thus, a strain-sensing mechanism would not give the same output as a stress-sensing mechanism. Recently a “stress-sensing vs. strain-sensing controversy” has been stirred up [see replies to Hamant et al. (2008) by Schopfer and Meyerowitz in science e-letters, and (Moulia et al., 2011) and (Hamant, 2013)]. Addressing this issue requires a further step in the modeling.

### From cellular mechanisms to quantitative local mechanosensing

#### Local mechanosensing of external loads: the “strain-sensing model”

**Figure 7:**
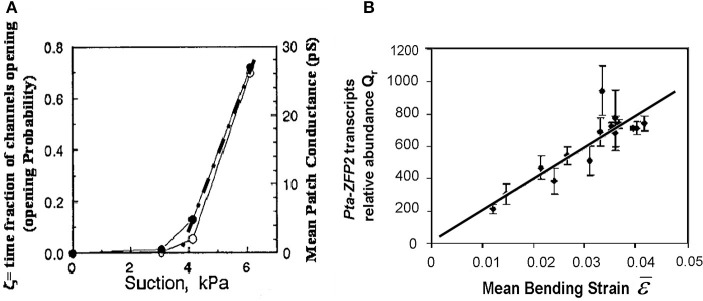
**Local mechanosensing of external loads. (A)** Probability of mechanosensitive channel (MsC) opening and mean patch conductance as a function of patch depression (and hence membrane tension and MsC strain). Open and filled circles, two replicates. Dashed dotted line, linear fit. Modified from Ding and Pickard (1993), Copyright# 1993, The Plant Journal, John Wiley and Sons. **(B)** Relationship between the relative transcript abundance Qr of the primary mechanosensitive gene *Pta ZFP2* (measured by Q-RT-PCR) and predictions from the Strain-Sensing model through the volume-averaged strain in the bent stem segment ε, (i.e., Sum of the Strain-Sensing normalized to the volume of the bent tissue; Coutand et al., 2009, Journal of Experimental Botany, by permission of the Society for Experimental Biology).

Among the mechanisms involved in mechanosensing, mechanosensitive ionic channels, often known as stretch-activated channels (SAC) have been the subject of detailed quantitative studies using the patch-voltage-pressure-clamp technique on protoplasts, cells enzymatically stripped of their walls (e.g., Ding and Pickard, 1993; Haswell et al., 2008). Altering the turgor pressure induces strains and tensional stresses in the plasma membrane and in the channel. The ionic current passing through the channels can be monitored after clamping the voltage, thus quantifying their mechanosensitive responses. The general shape of these response curves is sigmoidal, and can easily be linearized in the range of small strains (Figure 7A). Based on these results, we assumed that the local mechanosensitive function of a tissue element can be approximated through a linear function over a threshold (Coutand and Moulia, 2000; Moulia et al., 2011):

(10) $$dS_{i}=k_{s}\;\cdot \;\left(\varepsilon -\varepsilon_{0}\right).dV\text{ if }\varepsilon >\varepsilon_{0},{\text{else dS}}_{i}=0$$

where dS_*i*_ is the local signal in the cell (in Figure 7A, dS_*i*_ = dI, where I is the ionic current), k_*s*_ is a mechanosensitivity factor (*k*_*s*_ = 0 defines an insensitive tissue, while higher *k*_*s*_ values equate to greater sensitivity), ε is the local mechanical strain of the tissue element, ε_0_ is a possible strain threshold or minimal effective strain (ε_0_ ≥ 0) (see Moulia et al., 2006 for a review), and dV is the volume of the tissue element.

Equation (10) assumes that only tensile strains are sensed (ε > ε_0_ ≥ 0), but it can be extended straightforwardly to the case where both tensile and compressive strains are sensed in proportion to their absolute value, as is observed in animal bone tissues (Schriefer et al., 2005).

Equation (10) was assessed experimentally in *Populus tremula × alba* (*Pta*) (Coutand et al., 2009) by measuring the expression of the primary mechanosensitive gene *ZFP2*. *ZFP2* codes for a zinc finger protein that is transiently over-expressed as early as 5 min after straining in the strained tissues, probably in a cell-autonomous manner (Leblanc-Fournier et al., 2008; Martin et al., 2009, 2010) The response of the cell mechanotransduction pathway—from the initial reaction in the cytoplasm to primary gene expression in the nucleus—could thus be assessed by measuring Q_*r*_, the relative abundance of *ZFP2* transcripts in small slices of the stem using quantitative real-time PCR (Coutand et al., 2009). The bending stresses and strains are highly heterogeneous across a stem element. An integrative model was thus necessary to express the prediction of Equation (10) at the level of a stem segment and to assess it experimentally. Combining Equation (10) with the strain field equation in bending (Equation 4), it was possible to derive

(11) $$Q_{rorgan}=\frac{k_{s}.k_{ds}}{C_{0}}\cdot \bar{\varepsilon }-\left(\frac{k_{s}.k_{ds}}{C_{0}}\cdot {\bar{\varepsilon }}_{0}-1\right)=k_{r}\cdot \bar{\varepsilon }-\left(k_{r}\cdot {\bar{\varepsilon }}_{0}-1\right)$$

where Q_*r*_ is the ratio between the abundances of *Pta ZFP2* transcripts in the strained tissue elements and those in the unstrained control), k_*ds*_ is the sensitivity of the pathway downstream of the primary sensory reaction, C_0_ is the baseline transcript concentration in the unstrained control, k_*r*_ = k_*s*_ *k*_*ds*_/C_0_ is the apparent sensitivity of relative gene expression, and ε is the volume-averaged tensile strain (see Moulia et al., 2011 for details).

Our local mechanosensing model (Equation 11) thus predicts a linear increase in the relative expression of *ZFP2* with an increase in the mean strain ε, a prediction that can be tested experimentally. Indeed, the experimental relationship between measured Q_*r*_ and volume-averaged strain ε was found to be linear (Figure 7B), with Equation (11) explaining 77% of the 1:500 variation in Q_*r*_. This validates the hypothetical strain-sensing model stated in Equation (10) and gives the first *in planta* measurement of the mechanosensitivity of the mechanotransduction pathway. Under the conditions of this experiment, a 1% strain induces a transient 200-fold increment in transcription of *Pta-ZFP*2. It was surprising that the strain range in which this linear mechanosensing model holds true goes up to at least 5%, i.e., well beyond the range of elastic strains in cell walls. Cell internal components are likely to undergo a much larger range of elastic deformation than the cell wall alone, explaining the proportional sensing of strain even when wall stresses eventually plateau (see Sato et al., 2005).

#### Local mechanosensing of internal loads: cellular stress-feedback model

**Figure 8:**
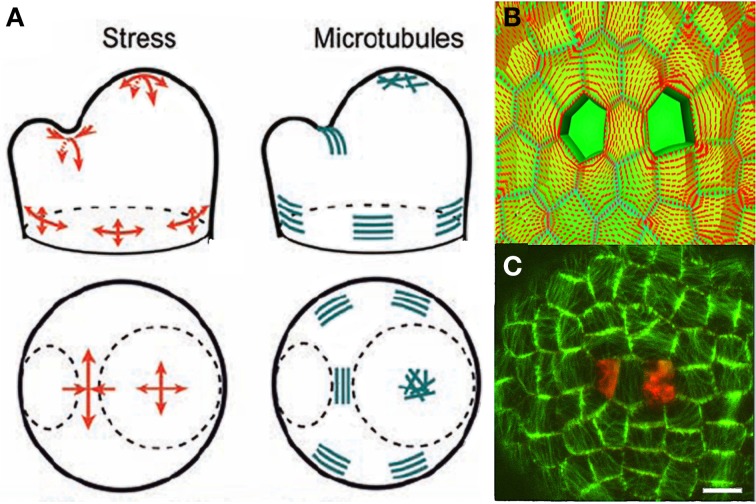
**Mechanosensing of internal loads in the SAM and microtubule re-orientation from Hamant et al. (2008), reprinted with permission from AAAS. (A)** Schematic representation of stress directions and microtubule orientations in the different parts of an SAM bearing a cylindrical primordium. **(B)** Principal stress pattern at the outer surface of the meristem simulated in an FEm of a patch of SAM at the top of the dome with a two-cell ablation. The stress pattern is circumferential to each of the ablated regions and stress alignment is enhanced in the cell between the two ablated cells. **(C)** Cortical microtubule distribution in the L1 layer in the central zone after a two-cell ablation as visualized by the expression of a construct fusing the Green Fluorescent Protein and the Microtubule Binding Domain (GFP-MBD), Scalebar, 5 μm.

The mechanisms underlying the responses to internal loads have been investigated much less than those involved in sensing external loads. Mechanical signals control (i) the amount and distribution of the active PIN1 auxin transporters, possibly though Ca^2+^ influx acting on PINOID proteins via TOUCH3 proteins (Heisler and Lam, 2010; Nakayama et al., 2012), and (ii) the alignments of cortical microtubules (CMT) and the orientation of cell division planes. The calculated stress pattern in the SAM outer L1 layer, and the CMT distribution determined experimentally (Hamant et al., 2008) were very similar (Figure 8A).

However, this observation is only correlative. A step forward was made when it was confirmed that the distribution of microtubules changed to match the redistribution of the wall stresses as predicted by the local FEm when the meristematic dome was compressed or when two holes were made in the L1 layer, (Figures 8B,C). However, this still did not provide a mechanistic link. A putative sensing mechanism may involve wall-associated protein complexes linking the cell wall to CMT that would then be directly subject to cell wall stresses, but there is no direct experimental evidence for this at the subcellular level (see Baluska et al., 2003; Landrein and Hamant, 2013 for discussion).

**Figure 9:**
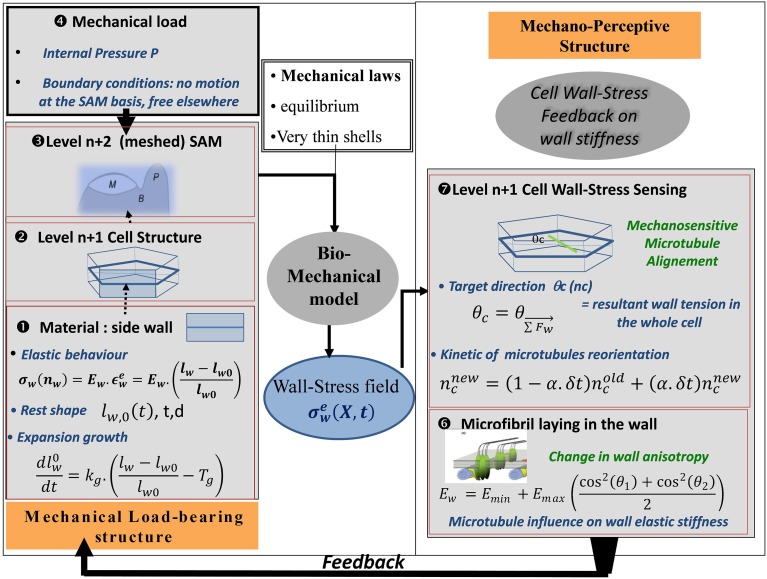
**Schematic representation of the SAM Stress Feedback model (SAM SFm).** The SAM SFm (Hamant et al., 2008) incorporates an ISM biomechanical model of the mechanical load-bearing structure of the SAM, and a mechanobiological model of the responses to the mechanical state of a cell in terms of (i) cell-wall stress sensing by CMTs and (ii) the consequences on the longitudinal elastic stiffness of the cell wall due to the direction of the laying down of cellulose microfibrils with respect to the longitudinal direction of the cell wall. The elemental brick of the biomechanical model is a piece of cell wall ❶ (called the cell-wall element) which displays two rheological behaviors: (i) elastic straining and (ii) expansion growth changing the rest length l_*w*,0_ of the element at a rate that is proportional to its elastic strain over a certain threshold. Cell wall growth is therefore analogous to visco-plastic creep. The transverse height (d) and thickness (t) of the wall element are assumed to be constant. Two levels of structure can then be assembled. At the first level, ❷ the side walls of a single hexagonal cell are assembled The model can be run at this level, giving rise to the cell-level formulation of the SFm. Otherwise the cells can be assembled to form a surface mesh with typical SAM geometry❸, in the SAM-level formulation of the SFm. The load is the turgor pressure of inner tissues considered to be fully borne by the L1 cell(s) ❹. This ISM biomechanical module outputs the (elastic) wall-stress field σ^e^_w_ at every position X on the different cell-wall elements composing the mechanical structure, at a given time, as well as the changes in rest-lengths of all the cell-wall elements due to expansion growth. This updates the geometry of the cellular structure for the next step and the outputs are transmitted to the mechanobiological module. In the mechanobiological module of the SAM SFm, the mechanosensitive step occurs at the level of the cell ❼, as it is an intrinsic cellular process. The central hypothesis of the module is that CMTs are re-aligned to the current direction of the direction of the resulting stress θc, but this occurs at a constant pace with only some of the overall CMT population (n_cnew_) being reoriented during each step (the rate is assumed to be independent of the mechanical state). The current mean orientations of CMTs in two cells sharing a given cell-wall (θ_1_(t), θ_2_(t)) determines the longitudinal elastic stiffness of the side cell wall E_*w*_ presumably through the orientation of the laying down of the new cellulose microfibrils with respect to the existing wall, changing the anisotropy of the cell wall elastic rigidity and hence its longitudinal stiffness (here assumed to be instantaneous) ❻ (note that (θ_1_(t), θ_2_(t)) may differ from the targeted orientations (θc_1_, θc_2_) specified by the stress-feedback as CMT reorientation takes time). The latter process occurs at the level of each cell-wall element so different walls of the same cell differ in the amount of elastic stiffening they undergo. The new value of wall elastic stiffness E_w_ is the output of the mechanosensitive module and is transferred to the mechanical module, in which the elastic stiffness is updated, immediately changing the constitutive law of the cell walls, and thus giving rise to a new mechanical equilibrium at the next step. This change in wall elastic rigidity is the way the current wall-stress state feeds back on the growth of the meristematic cells. Note that cell division may occur (not shown). A phragmoplast (new cell wall) is laid down parallel to the current CMT direction θ1 whenever the size of the stem passes a certain size threshold thereby changing the structure of the L1 cell wall “mesh.”

In order to assess this mechanism of microtubule alignment by wall stresses with respect to the data on SAM dynamics, a new model was needed. Microtubule reorientation takes 4–12 h, long enough for growth to occur. Thus, a model was required that included cell geometry, growth, mechanosensing of load distribution, and microtubule orientation. This model was called the Two-Dimensional Cellular Stress Feedback model (2D SFm, Figure 9; Supplemental Material SM2).

***Mechanical structure***. The material element of this model is a piece of cell wall, assumed to display linear elastic properties, i.e., its stresses are proportional to its strains. The coefficient of proportionality is the stiffness of the wall material E_*w*_, its Young's modulus. Only one-dimensional (1D) stretching of the wall is considered. E_*w*_ is under biological control, modeled as depending on the auxin concentrations in adjoining cells on both sides of the wall (see Supplemental data), and on microtubule orientation in the same cells (θ_*c*1_ and θ_*c*2_), according to:

(12) $$E_{w}=E_{min}+E_{max}\left(\frac{\cos^{2}\left(\theta_{1}\right)+\cos^{2}\left(\theta_{2}\right)}{2}\right)$$

E_*min*_ is the elastic stiffness of the isotropic cell wall matrix, and $E_{max}\left(\frac{\cos^{2}\left(\theta_{1}\right)+\cos^{2}\left(\theta_{2}\right)}{2}\right)$ is a stiffening term related to the directional angle Θ of CMTs (and hence microfibrils) relative to the wall direction on both sides of the cell wall. This cos^2^(Θ) angular dependency simply specifies mathematically the idea that parallel and antiparallel orientations both lead to the maximal longitudinal stiffening, whereas perpendicular orientation leads to no stiffening.

Interphasic expansion growth of the walls of meristematic cells is modeled by increasing the resting length of walls l_*w*0_ at an absolute rate that is proportional to the amount of elastic strain of the wall above a yield threshold. Cell wall synthesis is assumed to follow wall extension (and cell division). The wall elements are assembled into a two-dimensional tissue model representing the cells of the L1 layer as hexagonal boxes, only considering the lateral walls of L1 cells. Each wall is considered to act as a 1D spring carrying a force F_*w*_. The cell corners are assumed to behave like ball-joints in that there is no stiffness when the angle is changed. Finally, cell division is assumed to occur when cells reach a threshold size, and the new wall runs through the barycenter of the original cell and parallel to the direction of microtubules.

***Load modeling mechanical equilibrium***. Just as in the PVm, the load comes from the turgor pressure of the inner cells and is assumed to be homogeneous. This puts the L1 layer under 2D stress and the curved configuration allows local curvature to balance the inner force and the tensile reaction within the L1 wall.

***Mechanosensing and feed-back mechanisms***. The mechanosensitive reorientation of CMTs is modeled assuming that Θ_*c*_, the CMT direction for a cell, is sensitive to wall stresses. The model does not address the stress distribution within the wall-associated proteins and the cytoskeleton though. It is only assumed that the mean CMT orientation somehow follows the orientation of the net force resulting from the tensions in all the side walls:

(13) $$\theta_{c}=\theta_{\vec{\sum F_{w}}}$$

This alignment response is not instantaneous but occurs at a constant rate. This is the only mechanosensitive step in the model.

The feedback mechanism then comes from the following assumptions. The current mean CMT orientation is supposed to alter the elastic stiffness of each cell wall by modifying the direction of the cellulose microfibrils in the wall and hence the anisotropy of cell wall stiffness. This modifies the amount of growth in the different cell walls and the direction in which the new cell wall is laid down when a cell divides.

The inputs of the model are the turgor pressure, the shape of the L1 cells, and possibly the distribution of auxin. It predicts the elastic stresses and strains in the walls, the expansion growth, the mean orientation of CMT, and the orientation of phragmoplasts. These outputs depend on seven parameters (E_*min*_, E_*max*_, plastic growth extensibility, yield strain threshold for growth, the two parameters of auxin sensitivity, and rate of mechanosensitive reorientation of CMT, see Supplemental material for more details).

An important feature of this model is that different rates of expansion originate from the applied forces (pressure and wall-wall interactions), and the dynamics of the cell wall elastic stiffness. Hence, there is no explicit relationship between maximal stress and maximal growth direction in the model. Depending on the stress patterns, the model can predict maximal growth along the maximal stress direction and perpendicular to it.

The validity of this model could not be assessed experimentally at the local level, but it was included in a model of the mechanosensitive behavior of the whole SAM (Section Whole-organ integration and experimental assessment).

## Whole-organ integration and experimental assessment

### The sum of strain-sensing model, S^3^m

#### Quantifying global thigmomorphogenetic responses

To properly lay out the problem of integrated mechanosensing at this point, we need to consider the global growth responses of the plant to external bending loads in more detail. This has been made possible by using a quantitatively-controlled bending device while continuously monitoring primary elongation or secondary thickening using linear voltage displacement transducers (Coutand et al., 2000). It was found that elastic bending at the base of the stem induced a thigmomorphogenetic response in the distal primary growth zone, implying that a long-range internal secondary signal S_*i*,1_ traveled from the bent tissues to the responding primary tissues (Coutand et al., 2000; also Brenner et al., 2006). The propagation of this signal to the apex is much faster than the typical reaction time of growth responses, and there is no obvious damping over longer distances (Moulia et al., 2011). The nature of the carrier of this long-distance signal is currently being investigated. Given its velocity it could be either an electric signal in the phloem, or more likely a pressure pulse in the xylem (Lopez-Rodriguez et al., 2014; Tixier et al., 2014). In contrast, the secondary growth response seems local to the bent zone (Mattheck and Bethge, 1998; Coutand et al., 2009). For both primary and secondary growth responses, the initially growth stops for one to a few hours, then growth restarts and eventually the growth rate returns to that of unstimulated controls. For primary growth, the recovery time is highly dependent on the amount of bending strain applied, typically ranging from 100 to 1000 min. No compensatory growth is observed so at the end of the experiment bent plants are shorter than control plants (e.g., 2 mm shorter per bending stimulus in the experiment by Coutand et al., 2000). Secondary growth though shows clear and long-lasting growth stimulation after the initial inhibition, with growth rate increasing over 3 days then decreasing to the control rate over the next 3–4 days. The effect of this stimulation of secondary growth (+0.35 mm per bending stimulus) was approximately 30 times higher than the effect of the initial inhibition, resulting in an overall stimulation of radial growth. Unlike primary growth, the timing of the response was much less dependent on the amount of bending strain than on the peak (and total) increment in growth rate (Coutand et al., 2009, 2010).

#### Integrating local mechanotransduction into plant mechanosensing

**Figure 10:**
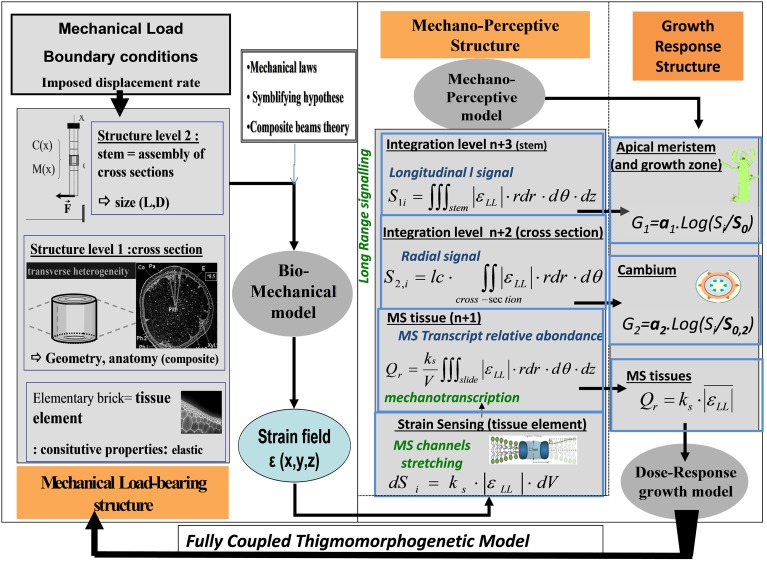
**Schematic representation of thigmomorphogenetic model including the ISM beam model and the S^3^m.** The ISM model of the mechanical load-bearing structure (left) is the CBmS designed by Coutand and Moulia (2000) to analyze stem bending experiments (see Figure 3). It is based on a validated composite-beam model of plant organ flexion (Moulia and Fournier, 1997). In its most simple configuration its inputs are the curvature field *C*(ζ) and the bending moment *M*(ζ) along the stem (measured as in Moulia et al., 1994). Its parameters are: (i) length, L, and diameters along the stem, *D*(ζ); (ii) estimates of tissue stiffness (longitudinal Young's moduli Coutand and Moulia, 2000); and (iii) the anatomical cross-sectional images processed using the model by Moulia and Fournier (1997). The elementary unit is a piece of tissue assumed to behave in the linear elastic range. Like all models based on beam-theory, this model defines two integration levels: the cross-section (which can be heterogeneous) and the stem. From the curvature field, it computes the strain field, ε(*x*, *y*, ζ, *t*) (and the stress field σ(*x*, *y*, ζ, *t*) if required) with ζ being the position along the stem and *x,y* the coordinates within the current cross-section of the stem, and t the time. The mechanosensitive model is the S^3^m model (Moulia et al., 2011). Its inputs are the strain fields ε(*x*, *y*, ζ, *t*) in each stem, and the stem geometry factors L and D(ζ), (all these data are received from the ISM beam model). S^3^m then generates the local sensing in a tissue element, and, if needed, the predicted amount of transcription Qr of a primary mechanosensitive gene. Then the integrated secondary signals S_*i*,1_ and S_*i*,2_ reaching the primary and secondary meristems, respectively, are computed. These signals are inputs of a module of thigmomorphogenetic growth responses (Coutand and Moulia, 2000; Coutand et al., 2009) outputting logarithmic dose-response modulations of primary and secondary growth. In a fully-coupled dynamic model of thigmomorphogenesis, the outputs of the thigmomorphogenetic growth response module can be used to update the size and geometry of the stem at the next step, so time integration can be simulated.

Why do stems of different shape and structure respond differently to the same external load? We aimed to assess whether the strain-sensing hypothesis, combined with structural and geometrical effects on load distribution across the stem structure, can explain the variability in plant responses (Coutand and Moulia, 2000; Coutand et al., 2000, 2009). For this, we set out to build a *minimal model* of mechanosensing integration, from the level of the strained tissue element up to the thigmomorphogenetic growth responses in the entire stem. This model has been called the Sum of Strain-Sensing model (S^3^m). Its development involved several steps (Coutand and Moulia, 2000; Coutand et al., 2009) and it was only completely assembled a few years ago (Moulia et al., 2011). This model is designed to chart the effects on the global thigmomorphogenetic responses of both the mechanical and the mechanoperceptive structures of the organ. The model was made with the purpose of competitively assessing two possible candidate mechanisms for the mechanosensing of external loads, strain-sensing vs. stress-sensing. Building the model was analogous with the process of integrative modeling in structural mechanics as illustrated in Figure 10 (presented in detail in the supplemental data), but extended it to include purely biological sensory responses.

The starting point was the local strain-sensing model (Equation 10) which states that the secondary signal output of each cell, dS_*o*_, is proportional to the mechanotransduced signal in the mechanostimulated cell, and hence to dS_*i*_ (hypothesis H1).

The long-distance signal propagation was very fast compared with the growth response and was not damped down, so it could be neglected. The simplest model for the integration of the mechanical sensing is that the output signals, dS_*o*_, of all the mechanosensitive cells simply sum up into a global secondary internal signal S_*i*_ (hypothesis H2). In short, for a given strain amplitude, the more cells that are strained, the higher the S_*i*_ is.

Subapical primary growth responds to distant sensing throughout the stem volume Vs. The internal signal propagated axially along the whole stem and controlling the response of primary growth S_*i*,1_ can then be written as the sum total of all the local signal outputs from the strained cells across the stem volume Vs (Coutand and Moulia, 2000):

(14) $$S_{i,1_{\left(\varepsilon \right)}}=\underset{V_{s}}{∭}k_{o_{\left(\varsigma ,y,z\right)}}\cdot \;\left(\varepsilon_{\left(\varsigma ,y,z\right)}-\varepsilon_{0}\right).dV$$

where ζ is the distance from the apex and (*y, z*) describes the position of the tissue elements across the cross-section of the stem and the triple sign means the sum along the three dimensions of the stem volume.

By analogy the thigmomorphogenetical signal controlling secondary growth S_*i*,2_ can be computed as the sum of the elementary signals dS_*o*_ on a one-cell thick cross-section (see Figure 10).

As can be seen in Equation (14) the mechanosensitive structure of the plant (at a particular time) is given by the spatial distribution of mechanosensitivity *k*_*o*(ς, *y*, *z*)_ and thresholdε_*o*(ς, *y*, *z*)_. However, comparative tests of the S^3^m have shown that the most determinant factor was the geometry of the stem. For simplicity, more recent studies took mechanosensitivity to be homogeneous over all tissues (e.g., Coutand et al., 2009). If *k*_*o*(ς, *y*, *z*)_ and ε_*o*(ς, *y*, *z*)_ are constant, then they can be factorized in the spatial integrals, so that the model for the control of primary growth becomes

(15) $$S_{i,1_{\left(\varepsilon \right)}}=k_{o}\left(\underset{V_{s}}{∭}\varepsilon_{\left(\varsigma ,y,z\right)}.dV\right)-k_{o}\varepsilon_{0}.V_{s}=k_{o}S_{\text{1strains}}-\Sigma_{0}$$

where *S*_1strains_ is the integrated stimulus summing all the strains of the cells and Σ_0_ is the integrated minimal effective strain. The same expression can be used for secondary growth.

This model thus predicts that the integrated signals reaching the two meristems are linearly dependent on integrals of the strain field over the domains of mechanosensitive integration for primary and secondary growth (S_1strains_ and S_2strains_, respectively).

This prediction was tested quantitatively against the corresponding growth responses described earlier. Compared with what we studied for the local mechanosensitive gene expression (Equation 11 and Figure 7B),the prediction of S^3^m is no longer that the growth response should be linear with S_i,strains_ but that the growth responds in a dose-dependent manner to the sum of strains S_i,strains_. Another way of thinking about this is that collecting more cells in the strained tissues for the analysis necessarily entails adding RNA to the sample (linearity), but the biological thigmomorphogenetic response of growing tissues to the supposed integrated signal S_*i*_ may not be additive.

**Figure 11:**
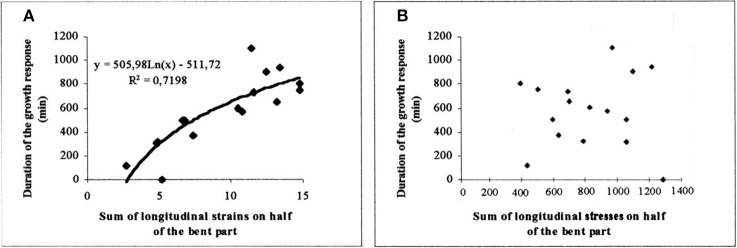
**Experimental assessment of the S^3^m model.** Dose-response curve of the recovery time of the primary growth response after bending plotted against the candidate internal signal (S_1,strains_) predicted by the S^3^m model (adapted from Coutand and Moulia, 2000 Journal of Experimental Botany, by permission of the Society for Experimental Biology). **(A)** A logarithmic relationship is obtained under the hypothesis that the mechanosensed variable is the strain and which explains 72% of the overall response. **(B)** No relationship is obtained under the hypothesis that the mechanosensed variable is the stress. —, log fit; ♦, experimental results.

As shown in Figure 11A, a tight logarithmic relation was found between the primary growth response (recovery time τ_r_) and S_1strains_ which explains 75% of the 1:10 variation in the response (Coutand and Moulia, 2000).

(16) $$\tau_{recovery}=a_{1}.\ln\left(\frac{S_{1_{\text{strains}}}}{S_{0_{\text{1strains}}}}\right)\text{ for }{\text{S}}_{\text{1strains}}>S_{0_{\text{1strains}}}$$

where a_1_ is the global thigmomorphogenetic sensitivity of a plant (including both the initial sensitivity of the mechanoperceptive structure of the plant *k*_*o*(ς, *y*, *z*)_ and the responsiveness of the meristem to a long distance signal S_*i*,1_) and *S*_0 _1strains__ is the threshold over which meristematic cells perceive the global systemic signal reaching the growth zone. (Note that *S*_0_1strains__ is not the same as the integrated minimal effective strain threshold Σ_0_ presented earlier).

Similarly, a relationship was found between S_2strains_and the radial growth response, which again explains 75% of the 1:5 variation generated by varying stem bending with different stem sizes (Coutand et al., 2009). An initial experiment on poplar suggested that a linear relationship between the radial growth response and S_2strains_ was statistically slightly more significant than a logarithmic relationship. However, analysis of a set of dicot tree species (Coutand et al., 2010) showed that the logarithmic relationship is more generic (also see Telewski, 2006).

It should be noted that using S^3^m the global thigmomorphogenetic sensitivity of a plant can be described quantitatively using just two parameters for the primary growth response (a_1_, *S*_0_1strains__) (Coutand and Moulia, 2000) and two for the secondary response (a_2_, *S*_0_2strains__) (Coutand et al., 2010). Varying the load and/or plant size and anatomy affects the S_1strains_ value along the x-axis in Figure 9, and thus the value of the response, but the relationship expressed in Equation (16) (and the corresponding log response curve) are invariant. This relationship and the a_1_ parameter in Equation (16) are thus independent of both load intensity and plant size/structure.

Equation (15) involves an explicit integration of the effect of the mechanical and perceptive structures of the plant through the S^3^m model, a model that has been validated experimentally. This is not the same as a purely correlative dose-response curves with an “arbitrarily chosen” measure of the stimulus (e.g., force Jaffe, 1980a).

Finally, and very interestingly, we modified the local sensing equation so that local stress was the sensed variable instead of local strain. In this Sum of Stress-Sensing model, the 1:10 variation in the response was no longer explained, clearly disproving the stress-mechanosensing hypothesis for the control of growth by external mechanical loads (Figure 11B).

### Integrative stress-sensing model in SAM subject to internal loads

The SFm of a cell has been assembled into a cellular network encompassing a realistic SAM shape to simulate the entire dynamics of meristematic growth and morphogenesis, including primordial bulging, phyllotactic patterning, and distribution of CMT orientation. This integrative SAM Stress Feedback model (SAM SFm) was derived from an existing model relating auxin transport to phyllotactic dynamics (Jonsson et al., 2006). To account for a realistic distribution of growth rate across the meristem an *ad-hoc* dependency of the growth extensibility parameter k_*g*_ on distance to the center of the SAM was included (thereby introducing an 8th parameter, while artificially constraining the possible dynamics of the model).

**Figure 12:**
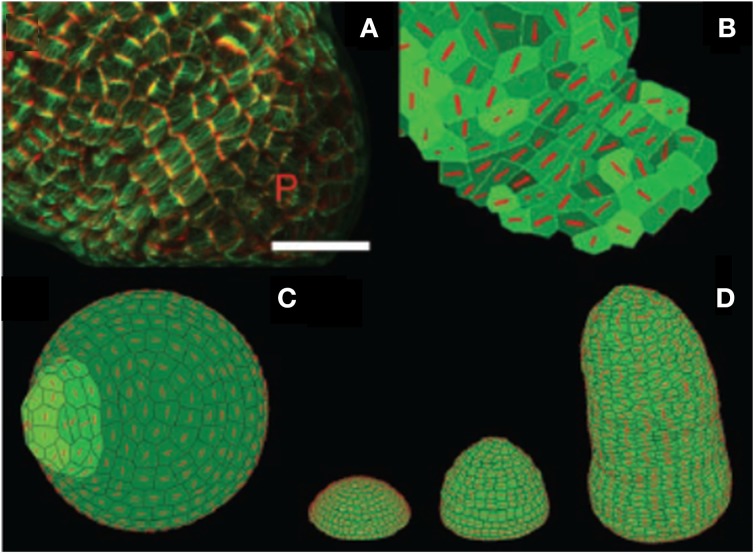
**Experimental assessment of the 2D Stress Feedback model (SFm) of the entire morphogenetic dynamics of the SAM from Hamant et al. (2008), reprinted with permission from AAAS. (A)** marking cortical microtubules (green) and cell shape (red) at the surface of a meristem generating a young primordium (P). Cortical microtubule marking is obtained using the expression of a fusion protein involving the Green Fluorescent Protein and the Microtubule Binding Domain (GFP-MBD) under the control of the constitutive promoter 35S (*p35S*::*GFP-MBD*) Scale bar, 20 mm. **(B)** Microtubule orientation (red bars) in cells in the 2D SFm (extracted from confocal data). Note the alignment of the virtual microtubule orientations in the boundary zone and compare to **(A)**. **(C)** Simulation of an auxin-induced primordium. The 2D SFm results in orthoradial alignment of microtubules around the growing primordium. **(D)** Tip-growing simulation with the stress-feedback model generating a growing stem. Microtubules align mainly orthoradially in the stem, which has a regular shape.

The overall dynamics of the model was qualitatively satisfactory. Moreover, as shown in Figure 12 the predicted CMT orientation was found to match experimental observation at the primordial boundaries, at the side of a growing primordium and at the stem-side base of the SAM. The robustness of the stabilization of CMT orientation during the formation of the primordial boundary and crease could be also assessed. Last but not least, an alternative Growth-Strain Feedback model (GSFm) for cellular mechanosensing was implemented and simulated within the same framework, and found to produce erroneous qualitative predictions (see replies to Hamant et al., 2008) by Schopfer and Meyerowitz in Science e-letters).

However, in these studies, the prediction by the SAM SFm could not be quantitatively assessed vs. the observed CMT reorientation. Growth-induced strains (and strain rates) were not measured concurrently, so the validity of the competing GSFm could not be really assessed. In other words, the GSFm might generate the wrong SAM dynamics not because its sensing hypothesis is wrong but because the modeling of growth and growth-induced auto-stresses is problematic. The elaborate task of testing the models was undertaken by Burian et al. (2013) who combined confocal measurement of CMT orientation dynamics with the sequential-cast (replica) technique, and careful comparative mapping of CMT orientation statistics, local surface curvature and strain-rates. First Burian et al. (2013) clearly disproved the GSFm and its central hypothesis that CMTs are always oriented perpendicular to the maximal growth. However, the analysis of the relationship between wall stress pattern and CMT orientation proved more complex. A priori two models were available to test for inferred stresses: the simple PVm (Figure 5), and the more complex numerical SAM SFm (Figures 9, 12). However, as seen before, using the numerical cellular SFm requires the estimation of eight parameters, as well as exact knowledge of the initial geometry of cells. This requires very complex, destructive and time-consuming measurements. Therefore, the authors relied on the much simpler PVm (Hamant et al., 2008) which only requires mapping of the curvatures of the L1 layer. This was achieved through the sequential replica method followed by three-dimensional (3D) reconstruction and differential geometry. Burian et al. (2013) found that the presumed geometry-derived stress distribution is not sufficient to predict CMT orientation throughout the SAM (i.e., other than at the primordium boundary), thereby rejecting the simple stress-feedback hypothesis. They argued that a better, qualitative match between estimated developmental changes in stress and CMT were found when mechanical auto-stresses derived from differential and heterogeneous growth were considered. However, a full assessment of this new hypothesis requires an extension of the numerical cellular SFm to include differences in pressure in the L1 layer, so that in-plane stressing between neighboring cells can be considered. The modeling of possible differential behavior of the inner and outer sides of the L1 layer may also be considered (as has been done in drosophila embryo models Supatto et al., 2005). This is likely to require substantial changes in the model such as a full coupling with water flows, and therefore many additional parameters entailing more experimental assessment of models.

### Major insights from the two examples of modeling

These two sets of work on the mechanosensitive control of growth and morphogenesis by mechanical cues have a lot in common. In both cases it was necessary to combine integrative models with guided experiments and to progress iteratively in a model≤ftrightarrowexperiment loop. The role of the models was to allow for the predictions of mechanistic hypotheses to be assessed experimentally both at the cellular and whole-organ scales. In both cases, ISM integrative biomechanical models were coupled with a mechanobiological model to study the load distribution, the mechanosensing, and the building of the overall growth and morphogenetic responses. This has allowed the clear definition of the three “superimposed” structures of the plant: the mechanical structure bearing and distributing the load, the mechanosensitive structure, and the responsive morphogenetic structure. The SAM could be considered a borderline case for this theoretical framework because there is very little visible tissue differentiation, so the three structures are merged (even though invisible dynamic patterning of cell fate is underway). On the contrary, the growing dicot stem offers a much clearer spatial and functional distinction between the three structures. The mechanical structure mostly involves the stiff tissues (although parenchyma does act as a filler and stabilizer, Gibson, 2013), the mechanosensitive structure is mostly parenchyma (and phloem), and the morphogenetic structures are the primary and secondary meristems. Therefore, changes in the geometry of the stem, and on the balance between the three structures may change the overall responsiveness of the plant to a given mechanical load (even if the intrinsic sensitivities of the mechanosensing cells and meristematic cells remain unchanged).

In all cases, the major insight is how the geometry of the organ influences both the load distribution and the amount of mechanosensing. Insightful dose-response curves cannot be obtained when only the overall mechanical load is considered (e.g., the force applied on the organ). In some experiments apparently good correlative dose-response curves with the overall load can be produced (e.g., Jaffe, 1980b), but this is because the three structures of the plant displayed very little variability, while there was a wide range of loads. Global dose-response curves risk causing confusion as they do not consider the mechanical and mechanobiological structures and processes involved in the interaction between the organ and its load and may be misleading when analyzing genetic variability of natural populations or mutant or stage variability.

The second insight brought by these two approaches is that the sensing is distributed, so that the relevant variable is the distributed change in mechanical state, i.e., stress or strain. It is now time to revisit briefly the strain-sensing vs. stress-sensing controversy (a more complete discussion can be found in Moulia et al., 2011). The conclusions of the two sets of studies outlined in this review are contradictory. However, these findings can be reconciled by postulating that different sensing pathways may be involved, each having a different mechanical relationship with the cell wall. Mechanosensing of external loads is thought to involve mechanosensitive ionic channels that sit in the soft cellular membranes and are gated by membrane tensional stresses (Haswell et al., 2008). They thus cannot sense wall stresses. But the membrane is more compliant than the cell wall by orders of magnitude and it is attached to and pressed against the cell wall, so it is forced to follow the straining of the wall. As a consequence the intrinsic stretch-activated-channels are stretched according to cell wall strains. On the contrary, cytoskeleton elements link to the cell wall at direct adhesion domains (Baluska et al., 2003). These linkers are partially embedded in the cell wall, so any cell wall stresses are directly transmitted to them. Moreover, unlike the surrounding polysaccharide wall, they are likely to behave elastically. So the change in their configuration, the strain, that ultimately modulates their biological activity (Monshausen and Haswell, 2013) is directly linked with elastic stresses within the surrounding wall. As these proteins are minute inclusions within the cell wall, their very local stress-strain pattern cannot be resolved from that of the surrounding cell wall, so the changes in their configuration are better predicted by the stresses they receive from the surrounding cell wall than by its global strain. This illustrates how important it is to precisely consider the cellular (and macromolecular) structures involved, and how strain and stress patterns propagate, not only through tissues and cells, but ultimately in the apoplasm and symplasm. This is a new domain in which modeling will proved a very valuable tool.

## Biomechanical and mechanobiological models for plant mechanosensing: from the plant in its environment to genes and back

### Biomechanical models as a tool to design and analyze experiments

We now review more systematically some of the many ways in which biomechanical and mechanobiological models have been used to gain insights into the mechanosensitivity of plants in their natural environments.

#### Mechanical models as state observers estimating stress and strain distributions

Mechanical models are almost indispensable tools for tracking how the mechanical loads are distributed throughout the organ or plant, scaling down to the mechanosensitive cells themselves. Indeed the load acts through the whole organ or plant structure, with possible mechanical focusing and amplification at specific places (related to lever arms, holes and curvatures). The stress distribution in the SAM (Figure 5) is primarily prescribed by the geometry of the dome and its connection with primordia, the saddle-shape of the crease having a huge effect. If one stem has a diameter that is twice that of another stem, when they are both submitted to the same bending moment M (Figure 4), the strains will be 8-fold higher in the more slender stem. If the two stems have same diameter the Young's modulus of one is twice that of the other, the strains in the stem with the lower Young's modulus will be only twice as high. Without a proper understanding of these effects a mechanobiologist can easily misinterpret experimental data. The case of stresses is particularly compelling and indeed, as already emphasized, stress is not an observable or measurable quantity. Engineers and mathematicians create models that act as “state observers” for the very purpose of inferring stresses in a structure (Villaverde and Banga, 2014). Interestingly though, a model to estimate stress distribution can be rather simple, as long as the equilibrium configuration of the organ and its mechanical load can be measured with sufficient accuracy and the organ is not rheologically too heterogeneous. In this case, the stress field can be calculated by applying the law of mechanical equilibrium. This was done in the PVm by Hamant et al. (2008). However, as soon as tissue patterns and/or growth-induced stresses among cells [also known as residual stresses or auto-equilibrated stresses, see (Moulia and Fournier, 2009; Moulia, 2013) for definitions] are involved, the picture becomes far more complex (Burian et al., 2013). Models then also need to consider strains and growth. Both elastic and growth-induced strains are observable quantities that can be measured even with non-contact techniques as long as the organ is accessible (e.g., Silk and Erickson, 1980; Barbacci et al., 2013, 2014). However, only models can give insights into the full distribution of strains (and strain rates) across the tissues and their dependency on the structure of the plant and the load. These models acting as strain-state observers (such as the CBmS) are more advanced than stress-state observers, as they handle the changes in configuration of the organ structure under the load as well as the reference length of its constitutive elements, their changes through growth (e.g., Barbacci et al., 2013), and other visco-plastic effects.

#### A guide for experimental strategies

We have seen that stem geometry has a major influence on the strains resulting from bending One problem that biologists face is that plants are never uniform. When studying thigmomorphogenesis, the classic experimental approach has been to apply a range of forces to “standardized” sets of plants. However, we now know that force is not the perceived variable. Therefore, a more appropriate strategy is to apply a range of strains in a controlled manner. The natural heterogeneity of stem diameters is a natural source of continuous variation in applied strains. We can therefore, take advantage of the two sources of variation in the amount of strain, the load, and the size of the plant. This strategy was used to disentangle the “strain-sensing vs. force-sensing” issue (Coutand and Moulia, 2000; Coutand et al., 2000). It is important to use appropriate controls as the size variations may confer other biological properties that produce confounding effects.

This experimental strategy can even be extended to study plants growing in windy conditions outdoors. In natural conditions, wind cannot be controlled easily. If the aim is to study a range of strains due to wind, using standardized plants will make the experimental design completely dependent on wind velocity so it can take a long time to accumulate the data on the desired range of strains. However, if plants with different geometries are chosen, the natural variability in geometry will bring a range of vibration frequencies and a range of strains for any given wind velocity. To choose suitable plant geometries, a mechanical model is required (Rodriguez et al., 2008; Sellier et al., 2008). The model would also give insights into the strain field, so the positioning of detectors measuring growth responses can be optimized.

Finally the wind-plant interaction model can be coupled with S^3^m (instead of the static bending mechanical model CBmS in Figures 4, 10) to test thigmomorphogenetic hypotheses on plant development outdoors.

### A toolbox for mechanistic systems biology

While mechanical and biomechanical models are instrumental in mechanobiology, hypothesis-driven plant mechanobiology can also benefit from well-based models.

#### A tool for handling scale changes in integrative biology

Throughout this review, we have emphasized that models are necessary tools whenever effects of changes in organizational levels and scales are involved. Considering that the cell is the structural unit of life, it is intuitive to ask whether the cell with its constitutive molecules should be the usual scale for modeling. A central insight from biomechanical and biological modeling is that there is no absolute need to burrow down to the macromolecular or even cellular scale, even when cellular mechanobiological responses are the object of the study (e.g., Hamant et al., 2008). This is certainly true for mechanical models. For example, both the CBmS and the PVm do not specify cells as finite elements, but they can still be used to understand the distribution of stress and strain in the apoplast, mechanosensitive cytoskeletal remodeling, and even gene expression. The SFm does not really consider the biological cell as the structural units as the inner mechanical structure of the cell, the cortical cytoskeleton, the transverse actin stress fibers, the vacuole, the nucleus or the endoplasmic reticulum are not considered. The stress and strain distribution through the cell ultrastructure is unknown. The model is simply a honeycomb-like apoplastic structure. Models of the inner structure of the cell are only necessary when the localization of the intracellular mechanosensitive responses are being studied, namely the precise molecular mechanism of mechanoreception (e.g., mechanosensitive channel opening or anisotropic cytoskeleton disassembly). The internal mechanical structure of the cell protoplast, and its links with the cell wall, is beginning to be revealed. Some models of animal cell ultrastructure have been built (Nick, 2011; Asnacios and Hamant, 2012), but they rely on simplified representation of the cellular structures (e.g., microtubule are modeled as cables). Detailed 3D molecular mechanical models are only feasible when studying the molecular dynamics of isolated macromolecules or oligomolecular complexes for which the crystallographic structure is known (e.g., a mechanosensitive channel in a patch of lipid bilayer, Sotomayor and Shulten, 2014). Another viewpoint is that plasmodesmata form a cytoplasmic continuum in tissues for mRNA trafficking between cells, so the cell is not the intuitive unit for mechanotransduction in plants. Finally, quantitative functional genomics has shown that master transcription factors may be less concentrated than their respective promoter sites in a given cell. In this context, transcriptional regulation at the single cell level may have a highly stochastic component that only becomes deterministic at the scale of a tissue element containing many interacting cells (Elowitz et al., 2002; Gandrillon et al., 2012).

#### A Tool for Genetic Dissection

Thigmomorphogenetic responses have been described in many plant species and it is interesting to compare responses between species. The responses may be different because (i) the level of applied strain is different, for example, due to the geometry of the different species, (ii) the species sensitivity to mechanical loading is different, or (iii) both. The sensitivity must be an intrinsic character of the species and it must be independent of plant size. From the S^3^m model we know

(17) $$\begin{array}{l} \frac{dR}{dt_{\max}}=a_{2}.\ln\left(\frac{S_{2_{\text{strains}}}}{S_{0_{\text{2strains}}}}\right)=a_{2}.\ln\left(S_{2\text{strains}}\right)\\ \;-a_{2}.\ln\left(S_{0_{\text{2strains}}}\right)\text{for }{\text{S}}_{\text{2strains}}>S_{0_{\text{2strains}}} \end{array}$$

Therefore, by applying similar ranges of *S*_2strains_ and measuring the daily increment in diameter of different species, we can plot the growth response against the logarithm of the applied *S*_2*strains*_, and fit a regression line in which the slope (a_2_) is the intrinsic mechanosensing sensitivity of the species and the intercept can be used to estimate the mechanical signal threshold (*S*_0_2strains__).

**Figure 13:**
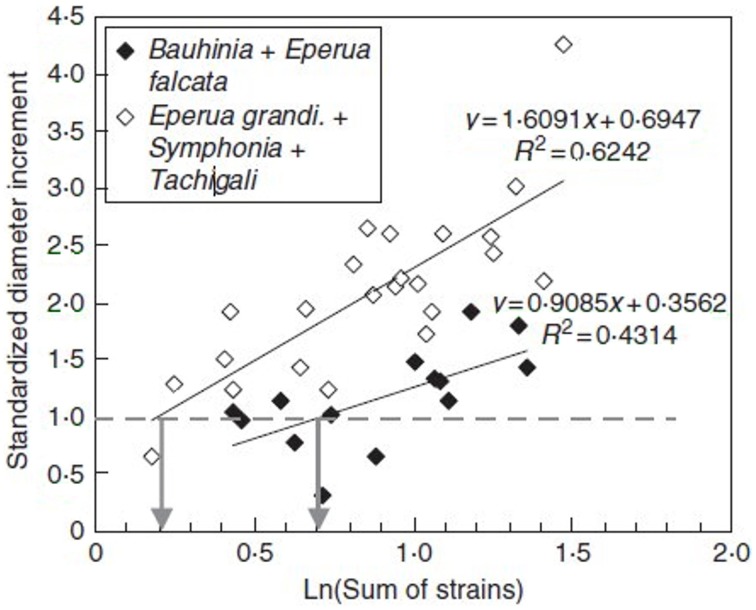
**S^3^m-assisted dissection of natural genetic diversity in mechanosensing.** Relationship between the standardized diametric growth responses of five neotropical forest species and the log of the candidate internal signal (S_2,strain_) predicted by the S^3^m model. S_2,strain_ = sum of strains over the cross-section during the bending treatment from Coutand et al. (2010), Annals of Botany, by permission of Oxford University Press.

The values of parameters a_2_ and S_02_ can be compared to see if there is any variability in mechanosensing between species. This conceptual framework has been used to study the mechanosensing variability between five sympatric tropical tree species (Coutand et al., 2010). No variability in mechanosensing sensitivity was found but differences in signal thresholds were found (Figure 13). Note that this conceptual framework could also be used to compare mechanosensing sensitivity between organs for example.

#### Enabling model-assisted phenotyping

The previous genetic study can be seen as an example of model-assisted phenotyping in which the S^3^m combined with a fairly simple experiment was used to extract two intrinsic parameters describing the mechanosensitivity of a species. The same approach can be used to phenotype collections of mutants or varieties. Defining intrinsic characteristics and enabling model-assisted high-throughput phenotyping is becoming a major challenge of systems biology (Bastien et al., 2013).

#### Identifying rewarding molecular studies

Another interesting heuristic property of integrative models is that they can help to identify (i) which modules are more influential than others in the global mechanobiological response, and (ii) which are insufficiently understood. This can help to guide molecular studies of gene regulation, for example, so that the most rewarding projects are designed. Genes targeted in this way are likely to trigger particularly significant phenotypes, and identifying key regulators significantly would improve our understanding of the whole system response. An example is our recent study of thigmomorphogenesis in wind acclimation of plants, which clearly pinpointed a current deadlock in advancing our understanding of mechanosensitivity accommodation in response to successive bending (see Leblanc-Fournier et al., 2014).

### Understanding plant responses to mechanical loads in their natural environment

Ideally the ultimate goal of systems mechanobiology would be to understand how plants acclimate in their natural environment. For example, for wind resistance, models are available to describe the mechanics of lodging, wind-throw and wind-break (see De Langre, 2008; Gardiner et al., 2008) for reviews), but existing growth models disregard thigmomorphogenesis so they do not deal with wind acclimation (Moulia et al., 2006). A major asset of the S^3^m is that it can be coupled with mechanical models to analyze the effects of the static and dynamic strains produced by wind-induced vibrations in plants (e.g., Gardiner et al., 2008; Rodriguez et al., 2008; Sellier et al., 2008). On the other hand, as S^3^m can also handle growth responses to wind, it can be coupled with structure-function growth models (see Moulia et al., 2006; Fourcaud et al., 2008 for general discussion). The overall wind resistance of plants due to genetic variation in intrinsic mechanosensitivity could be assessed in various (present, past or forecasted) climatic scenarii during *in silico* numerical experiments. There is obviously a lot of work to accomplish, but integrative mechanosensing models are surely a key breakthrough paving the way to a better understanding of the ecological and economic relevance of thigmomorphogenetic acclimation, for example, by exploring the consequences of global climate changes on stand growth and resistance to wind hazards.

## Conclusion: the challenges of systems mechanobiology

Plants respond to internal and external mechanical loads at many scales. The mechanical growth response can theoretically be broken down into four processes.

- Bearing the load, how the mechanical load is distributed by a plant structure.
- Sensing the effect of the load distribution by mechanosensing of local mechanical states.
- Transducing the signal through mechanoreceptor pathways to alter the expression of a specific set of transcription factors.
- Responding by the global retuning of growth rate and direction.
- However, none of these processes are disconnected from one another. The challenge of the integrative biology of mechanosensing is to operate across scales and processes and understand outputs in terms of the overall syndrome of mechanosensitive growth responses and their adaptive relevance.

As this loop involves several organizational levels and scales plus a host of interactions, it cannot be handled without models, placing this challenge within the realm of systems biology (Tardieu, 2003; Moulia and Fournier, 2009; Traas and Moneger, 2010). The bottom line here is that systems biology modeling and cross-comparisons against data produced through suitable experimentation makes it possible to test hypotheses. If a combination of hypotheses cannot be worked out without calculus, modeling becomes an extension of the experimental method (Legay, 1997). Therefore, the mechanical models need to be adjusted whenever necessary, such as to cope with a different experimental set-up or to natural conditions.

These mechanistic models have to be evaluated on their outputs, on the performance of their component mechanistic modules (e.g., Coutand et al., 2009), and their capacity to reflect natural genetic variation (e.g., Sierra-De-Grado et al., 2008; Almeras et al., 2009; Coutand et al., 2010). To be useful however, models need to be kept simple and amenable enough to use. Multiplying the number of elements and degrees of freedom makes models more difficult to analyze and assess experimentally. This difficulty may be partly circumvented by implementing a clear modular design and explicitly setting up organizational levels. Additionally, the development of non-destructive bio-imaging and micromechanical techniques (see references in Milani et al., 2013; Moulia, 2013) are ways to gather more information to precisely assess the modules. But there are still some intrinsic limits to model complexity if the model is to be used to assess mechanobiological hypotheses and/or to capture very non-linear behavior. Here mechanobiology diverges from standard mechanical engineering in which the model is mostly an attempt to assemble well-established physical laws into a given structure, not a way to assess a biological hypothesis. From this perspective, it is instructive that the SAM SFm with its eight parameters has yet to be assessed experimentally and that experimental progress has relied on the PVm. Hence, an effort to simplify models is called for (as is common in physics through dimensional analysis; see Rodriguez et al., 2008; Bastien et al., 2013, 2014) at the same time addressing them to specific hypotheses that can be experimentally falsified or upheld.

Overall, this strategy of combining heuristic models with experiments has provided many insights into the mechanosensitive control of growth. However, the complexity of the structural, dynamic, and regulatory aspects requires intense interdisciplinary work (Moulia, 2013). In particular, biologists need to become familiar with the key concepts of mechanics to collaborate productively with physicists and modelers in designing, criticizing, and experimentally assessing biomechanical and mechanobiological models. It is hoped that our review has convinced readers of the usefulness and reward of this interdisciplinary approach and made it more accessible. This interactive approach, including using modeling as a tool to extend the experimental method, contrasts with and complements alternative set-ups, for example, where experimentalists intensively collect data while bioinformaticians set up models and data mining programs.

The chosen examples considered in this review deal only with dicot growth, but the approach can be extended easily to monocots (with obvious adaptions to omit secondary growth responses), and even to most *Viridiplantae*. Again the examples all involved the shoot system, ranging from the cell to the organ level, but the methodology is in no way specific to these systems. Indeed there is a very active research making extensive use of integrative modeling to discover how root growth and morphogenesis is controlled by external and internal mechanical cues (see for example Ditengou et al., 2008; Bengough et al., 2011; Band et al., 2012; Jin et al., 2013). By the same token, works at the scale of the intracellular structures would be extremely useful. The way stresses and strains act inside the cell through the cell wall-membrane-cytoskeleton-nucleus continuum is yet to be fully grasped. The tensegrity model of the structural mechanics of the cytoskeleton has started to reveal unexpectedly how mechanical amplifications can take place through the cytoskeleton network (Nick, 2011), but the way it interacts dynamically with wall stressing and deformation remains to be elucidated (Hamant, 2013). Similarly, the responses of cell trafficking and plasma membrane straining and turnover upon cell stretching are very promising fields (Asnacios and Hamant, 2012; Nakayama et al., 2012). A more complete elucidation of the mechanosensitive gene networks controlling the cellular response is also a priority including how the cell division-cell elongation complex is modulated by mechanical cues. Finally this review has mostly focused on spatial integration, but the challenge of time integration is as important. When successive signals are applied to a living system, relative or absolute refractory periods of the mechanosensors and gene-regulated accommodation of the mechanosensitivity make it complicated to study the relationship between the signals and the system's responses (see Leblanc-Fournier et al., 2014). The analysis of the time aspect will also require two-way interaction between models and experiments.

The first successes of integrative mechanobiology of growth control have thus opened up a large set of questions for interdisciplinary research, which hopefully will elucidate many more aspects of the way plants have evolved to bear loads and remain stable when responding to internal and external mechanical challenges.

### Conflict of interest statement

The authors declare that the research was conducted in the absence of any commercial or financial relationships that could be construed as a potential conflict of interest.
