# Supplementary material for: Mechanosensitive control of plant growth: bearing the load, sensing, transducing, and responding
Source: Front Plant Sci. 2015 Feb 23;6:52. doi: 10.3389/fpls.2015.00052 (PMC4337334; doi:10.3389/fpls.2015.00052)
Supplement: Supplementary file 1 [file Presentation1.PDF]

Supplementary Material

Mechanosensitive control of plant growth: Bearing the load, sensing, transducing and responding

B Moulia [1,2¤]*, C Coutand [1,2¤], J L Julien [2,1]

[1] INRA, UMR 547, PIAF, F-63039, Clermont-Ferrand, France
[2] Clermont Université, Université Blaise Pascal, UMR 547, PIAF, BP 10448, F-63000, Clermont-Ferrand, France

¤ These authors contributed equally to the drafting of this review

*** Correspondence:** Dr Bruno Moulia, UMR PIAF Integrative Physics and Physiology of Trees, INRA, 5 chemin de Beaulieu, F-63039, Clermont-Ferrand Cedex 02, France

bruno.moulia@clermont.inra.fr

1. Supplementary material: a more detailed specification of the models

1.1. Composite beam model of the stem under external flexural load

1.1.1. Mechanical equilibrium

The applied force and the stress are linked through equilibrium: the external applied bending moment of amount FL must be equal to the sum over the cross section of internal moments resulting from the stresses σ_{LL} times the area element to which they apply (dS), time the internal lever arm to the neutral line y :

$$M = FL = \iint_S y\sigma_{LL} dS = \iint_S yyCE_{LL} dS = C\iint_S y^2 E_{LL} dS$$

Note that when the displacements are small the lever arm L can be computed in the unloaded state, if displacements are large, the lever arm must be computed in the loaded state at equilibrium.

In the simple case in which the cross section is made of two tissues of material stiffnesses E_{soft} and E_{stiff} arranged into three concentric layers (see figure Composite beam bending), the material stiffness can be factorized outside the sum which gives :

$$M = FL = C\left[E_{soft}\iint_{S1} yydS + E_{stiff}\iint_{S2} yydS + E_{soft}\iint_{S3} yydS\right]$$ where $\iint_S yydS$ is called the second moment of area (I).

For a cylindrical annulus, the second moment of area is equal to $I = \pi \frac{R_O^{\ 4} - R_i^{\ 4}}{4}$ where R_o and R_i are the outer and inner radii

Then $C = M / \left(E_{soft} I_1 + E_{stiff} I_2 + E_{soft} I_3 \right)$

The longitudinal strain can be computed by the product of considered position along the radius and the curvature by $\varepsilon_{LL} = yC = My / \left(E_{soft} I_1 + E_{stiff} I_2 + E_{soft} I_3 \right)$

And the longitudinal stress comes as

$\sigma_{LL} = E_i \varepsilon_{LL} = MyE_i / \left(E_{soft} I_1 + E_{stiff} I_2 + E_{soft} I_3 \right)$ where E_i is the Young's modulus of the tissue in which the stress is computed

1.2. **The Sum of Strain-Sensing Model S3m** (Coutand and Moulia, 2000;Moulia et al., 2011)

1.2.1. Local mechanosensing

The basal scale of the S3m model is an (homogeneous) piece of tissue called a tissue element.

When strained, this tissue element generates a local mechanosensing proportional to the amount of strain of that element and to its volume (this is the central hypothesis H1 of the S^3m model, that should be assessed experimentally).

$dS_i = k_s \cdot (\varepsilon - \varepsilon_0).dV$ if $\varepsilon > \varepsilon_0$, else $dS_i=0$ Eq S^3m-(1)

where dS_i is the local signal in the cell, k_s is a mechanosensitivity factor ($k_s = 0$ translates as insensitive tissue, while higher k_s equates to more sensing), ε is the local mechanical strain of the tissue element, ε_0 an eventual strain sensing threshold or Minimal Effective Strain ($\varepsilon_0 \geq 0$), and dV is the volume of the tissue element.

Eq S^3m-(1) assumes that only tensile strains are sensed ($\varepsilon > \varepsilon_0 \geq 0$), but also extends straightforwardly to the case where both tensile and compressive strains are sensed proportionally to their absolute value.

1.2.2. Mechanotransduction and gene expression

We may extend Eq S^3m-(1) to the entire mechanotransduction pathway, by stating that the increment of the content of transcripts of a primary mechanosensitive gene in a strained tissue element is also proportional to the initial local mecanosensing :

$$n_{t(\varepsilon)} - n_{t(0)} = k_s.k_{ds} \cdot (\varepsilon - \varepsilon_0).dV$$

i.e. $n_{t(\varepsilon)} = k_s.k_{ds} \cdot (\varepsilon - \varepsilon_0).dV + C_0.dV$ Eq S3m-(2)

where $n_{t(\varepsilon)}$ in the content of transcripts in the strained tissue elements, $n_{t(0)}$ the content of an unstrained control $n_t(\varepsilon=0$ k_{ds} with being the sensitivity of the pathway downstream the primary sensory reaction, and C_0 is the transcript concentration in the unstrained control (or baseline concentration),

In many cases the measurement of gene expression through Q_RT-PCR are presented as a relative expression ratio Q_r . Q_r is the ratio between the content of transcripts in the strained tissue elements $n_t(\varepsilon)$ and the content of an unstrained control $n_t(\varepsilon=0)$ (an eventual correction for similar volume in both samples is achieved through a multiplicative dilution prefactor, estimated through the assessment of the reference gene(s), not shown here).

The predicted Q_r in the element of tissue, noted $\hat{Q}_r$, should lead to

$$\hat{Q}_r = \frac{n_{t(\varepsilon)}}{n_{t(0)}} = \frac{k_s.k_{ds}}{C_0} \cdot (\varepsilon) - \left(\frac{k_s.k_{ds}}{C_0} \varepsilon_0 - 1 \right)$$

Moreover, the sample collected has a finite volume V (often much bigger than one cell). And, as seen earlier, when the stems are strained though bending, the strains are not uniform across the cross-section. Thus, equation 11.3 cannot apply directly: volume-averaged strain-sensing have to be considered. If we call $N_t(\varepsilon)$ the total number of transcripts in the volume of strained tissue analyzed, and $N_t(\varepsilon)$ the content of an unstrained control $N_t(\varepsilon)$ over the same volume V, then both are the sum of the contents of all the tissue elements over the volume V

$$N_{t(\varepsilon)} = \iiint_V k_s.k_{ds} \cdot (\varepsilon - \varepsilon_0).dV + \iiint_V C_0.dV \qquad \text{Eq S3m-(3)}$$

If we assume that the mechanosensitivity of the cells, $k_{mt=}$ k_s k_{ds}, and the baseline transcript concentration, C_0 , are constant within the segment of organs under study (tissues of same age, and same history), then :

$$\hat{Q}_{r\,organ} = \frac{N_{t(\varepsilon)}}{N_{t(0)}} = \frac{k_s.k_{ds}}{C_0} \cdot \left(\frac{\iiint_V \varepsilon.dV}{\iiint_V dV} \right) - \left(\frac{k_s.k_{ds}}{C_0} \cdot \frac{\iiint_V \varepsilon_0.dV}{\iiint_V dV} - 1 \right)$$

i.e.

$$\hat{Q}_{r\,organ} = \frac{k_s.k_{ds}}{C_0} \cdot \bar{\varepsilon} - \left(\frac{k_s.k_{ds}}{C_0} \cdot \bar{\varepsilon}_0 - 1 \right) = k_r \cdot \bar{\varepsilon} - (k_r \cdot \bar{\varepsilon}_0 - 1) \qquad \text{Eq S}^3\text{m-(4)}$$

Where $\hat{Q}_{r\,organ}$ is the ratio between the contents of transcripts in the strained tissue elements and in an unstrained control), k_r =(k_s k_{ds} / C_0) is the apparent sensitivity for relative gene expression and $\bar{\varepsilon}$ the volume-averaged tensile strain, (see (Moulia et al., 2011) for details).

Eq S^3m-(4) predicts that if the hypothesis (H1) of strain-sensing is correct, then $\hat{Q}_{r\,organ}$ should be proportional to the volume-averaged tensile strain $\bar{\varepsilon}$ over a threshold, and with a slope being the apparent sensitivity for relative gene expression. This prediction can be (and has been) assessed experimentally (validating hypothesis H1 and equation Eq S^3m-(1)

1.2.3. Long range signaling

As mechanostimulation sparks signals to move out of the cell, it was assumed that the secondary signal output by each cell, dS_o, is directly proportional to the mechanotransduction signal over an eventual threshold – and hence to dS_i -(hypothesis H1'), and can thus be written as:

$$dS_o = k \cdot dSi = k_o \cdot (\varepsilon - \varepsilon_0).dV \qquad \text{Eq } S^3\text{m-(5)}$$

(with k_o=k*k_s*k_{ds})

The simplest model for the integration of the mechanical sensing is then that the output signals, dS_o, of all the mechanosensitive cells simply sum up into a global secondary internal signal S_i (hypothesis H2). In short, the more cells are strained, the higher the S_i (see Moulia et al. 2011 for a more complete argument).

However, the domains of mechanosensitive integration seem to differ between the responses of primary and secondary growth zones. Subapical primary growth responds to distant sensing throughout the stem volume Vs, whereas distributed cambial growth only seems to be affected by strain-induced signals propagating radially in the cell layer of the cross-section A_s (hypothesis H2)

The internal signal propagated axially along the whole stem and controlling the response of primary growth $S_{i,1}$ can then be written as the addition of all the local signals ouput from the strained cells (hypothesis H3) :

$$S_{i,1(\varepsilon)} = \iiint_{V_s} k_{o(\varsigma,y,z)} \cdot (\varepsilon_{(\varsigma,y,z)} - \varepsilon_0).dV \qquad \text{Eq } S^3\text{m-(6)}$$

where ζ is the distance from the apex and (y, z) describes the position of the tissue elements across the cross-section of the stem.

By analogy, the internal signal propagating along the stem radius and controlling secondary growth, $S_{i,2}$, in the cambium at a position ζ on the stem thus become:

$$S_{i,2(\varepsilon,\varsigma)} = \int_{lc} \iint_{A_s(\varsigma)} k_{o(\varsigma,y,z)} \cdot \left(\varepsilon_{(y,z)} - \varepsilon_0\right).dxdydz = lc \cdot \iint_{A_s(\varsigma)} k_{o(\varsigma,y,z)} \cdot \left(\varepsilon_{(y,z)} - \varepsilon_0\right).dydz \qquad \text{Eq } S^3\text{m-(7)}$$

where l_c is the typical length of an initial cell in the cambium (or, in a more practical way, the longitudinal length over which radial growth is measured, i.e. the size of the pad sitting on the stem for the experimental measurement of diametral growth e.g 1 mm).

Note that the distribution of mechanosensitive tissues defining the mechanosensitive structure of the plant (of volume V_s and cross-sectional area $A_{s(\zeta)}$ at position ζ on the stem) does not span the whole stem volume but only the mechano-competent tissues. More precisely, the mechanosensitive structure of the plant (at a given time) is given by the geometrical description of mechano-sensitivity $k_o(\varsigma, y, z)$ and threshold $\varepsilon_o(\varsigma, y, z)$, just as the mechanical structure of the plant is given by the spatial distribution of the mechanical properties (e.g. the longitudinal Young's modulus $E_L(\varsigma, y, z)$ and a yield threshold $\sigma_0(\varsigma, y, z)$).

In a first approximation, Coutand and Moulia (2000) assumed that lignified tissues could be considered non-sensing, and that all living tissues had similar mechanosensitivity k_o. Using *Jr- ZFP2 in situ* RNA hybridization as a marker of mechano-competence in walnut stems (*Juglans regia*), Leblanc-Fournier et al. (2008) found that the cortical and medullar parenchyma of stems (and to a lesser extent some phloem parenchyma cells) displayed marking. The stiffer epidermal cells, collenchyma, xylem and sclerenchyma did not, nor did the meristematic cambium (this lack of marking in cambial and epidermal cells is another argument in favour of a non-autonomous , long-distance mechanosensitive control of growth, as expressed in equations Eq S^3m-(6)and Eq S^3m-(7)). However, comparative tests on the *Sum of Strain-Sensing* model have shown that the model output only marginally depends on the detailed distribution of mechanosensitivity (Coutand and Moulia, 2000), at least in the range of anatomical variability displayed by plants from the same cultivar at the same growth stage. The most determinant factor was the geometry of the stem. For simplicity purposes, more recent studies then took mechanosensitivity to be homogeneous over all tissues (e.g.(Coutand et al., 2009)). If $k_o(\varsigma, y, z)$ and $\varepsilon_o(\varsigma, y, z)$ are constant, then they can be factorized in the spatial integrals, so that the model evolves to:

$$S_{i,1(\varepsilon)} = k_o\left(\iiint_{V_s} \varepsilon_{(\varsigma,y,z)}.dV\right) - k_o\varepsilon_0.V_s = k_o S_{1strains} - \Sigma_0 \qquad \text{Eq } S^3\text{m-(8)}$$

$$S_{i,2(\varepsilon)}(\varsigma) = k_o.\left(l_c \cdot \iint_{As(\varsigma)} \varepsilon_{(\varsigma,y,z)}.dydz\right) - k_o\varepsilon_0 l_c A_s = k_o S_{2strains}(\varsigma) - \Sigma_{0,2}(\varsigma) \qquad \text{Eq } S^3\text{m-(9)}$$

The model thus predicts that the integrated signals are linearly dependent on integrals of the strain-field over the domains of mechanosensitive integration for primary and secondary growth ($S_{1strains}$ and $S_{2strains}$). This is what prompted the original name for the "Sum of Strains" model (Coutand and Moulia, 2000). However, a more accurate name is the *"Sum of Strain-Sensing" model (S^3m)*, as it is not the strain that is summed but the output of strain-sensing by cells (Moulia et al., 2011).

1.2.4. Mechanosensitive growth responses to long range signals

There are no theoretical reasons driving the choice of one type of a specific formulation for the dose-response of the meristems. We retained the "Weber-Fechner law" (W-F law, Hypothesis (H4)) because it has been widely (though not always) observed in human and animal sensory physiology and in plant gravisensing (see (Moulia and Fournier, 2009)) . The W-F law states that "the change in a stimulus that will be just noticeable is a constant ratio of the original stimulus". In other words the increment in growth response upon an increment in the "Sum of Strain" signal is inversely proportional to the prevailing level of the signal, so that the apparent sensitivity of the response decreases hyperbolically with the prevailing level of mechanical stimulus. This tuning of the sensitivity of the response is termed 'accommodation' (Schriefer et al., 2005;Moulia et al., 2006), and it is likely to be of major adaptive value by avoiding overreactions to noise.

This can stated mathematically as:

$$dG = a_i.\left(\frac{dS_{i\,strains}}{S_{i\,strains}}\right) = \frac{a_i}{S_{i\,strains}}.\left(dS_{i\,strains}\right)$$

Where G is the growth response, $S_{istrains}$ is the sum of strain of the mechanisenstive structure signalling to meristem i (i ∈ {1,2}) , $S_{1strains}$ is the sum of strain of the mechanosensitive structure for the control of primary growth, and $S_{2,strains}(\zeta)$ the sum of strain of the mechanosensitive structure for the control of secondary growth (note that $S_{2,strains}$ depends on ζ the position along the stem) , and a_i the responsiveness of the meristem i to the " sum of strain" signal.

In an integrated form this yields a logarithmic relation with the "Sum of Strain" signal

$$G_i = a_i.\ln\left(\frac{S_{i\,strains}}{S_{0i_{strains}}}\right)$$

Where $S_{0i_{2\,strains}}$ is a threshold for the growth response of meristem i (i ∈ {1,2}) (not to be confused with the Minimal effective strain threshold of local mechanosensing)

For the primary growth response, the mechanosensitive response was shown to act on the time for recovery of the undisturbed growth rate $\tau_{recovery}$. Applying the W-F law then yields:

$$\tau_{recovery} = a_1.\ln\left(\frac{S_{1\,strains}}{S_{0_{1strains}}}\right) \text{ for } S_{1strains} > S_{0_{1strains}} \quad \text{Eq } S^3m\text{-}(10)$$

For the secondary growth response, the mechanosensitive response was shown to act on the daily rate

of diametral growth $\frac{\partial D}{\partial t}(\zeta)$, yielding :

$$\frac{\partial D}{\partial t}(\zeta) = a_2.\ln\left(\frac{S_{2strains}(\zeta)}{S_{0_{2,strains}}}\right) \text{ for } S_{2strains} > S_{0_{2,strains}} \quad \text{Eq } S^3\text{m-(11)}$$

Eq S^3m-(10) and (11) predict that the two mechanosensitive growth responses should display a log-shaped master-curve when expressing them as a function of $S_{i,strains}$ ($i \in \{1,2\}$). This prediction can be (and has been) assessed experimentally (validating hypotheses H2-H4). The global thigmomorphogenetic sensitivity of a plant can thus be described quantitatively using only two parameters for primary growth response (a_1, $S_{0_{1strains}}$) and two for secondary response (a_2, $S_{0_{2strains}}$) (Coutand et al., 2010). These two quantities are integrative "macro-characters". They include the whole in-plant signalling process, through an explicit and validated mechanical and mechanosensitive integration of the interactions between the plant and its mechanical environment (load). Varying load and/or plant size and anatomy affects the $S_{istrains}$ values, and thus the value of the response, but the relation expressed in equations Eq S^3m-(10) and (11) -and the corresponding log response curve- remains invariant. This relation is thus independent of both load intensity and plant size/structure (note that the sensitivity parameters a_i are independent of size, whereas, in the present form of S^3m, the $S_{0i_{2strains}}$ thresholds are not)

Eq S^3m-(10) and (11) can then be used to predict the thigmomorphogenetic modulation of growth.) They should not to be confused with purely correlative dose-response curves with an "arbitrarily-chosen" measure of the stimulus. Indeed, they involve an explicit integration of the effect of the mechanical and perceptive structures of the plant through the *Sum of Stain Sensing* model — a model that has been assessed experimentally. However they do not provide a full prediction of growth over time. To do so they need to be coupled with a growth model predicting growth as a function of other external factors (e.g temperature, light, drought …)

1.3. Stress-Feedback model SFm

The presentation of the model in this supplementary material has been organized by process (mechanical stresses and strains, meristematic growth, mechanosensing, feedback rather than by scales as in the text (see figure Wall Stress feedback model by processes). This alternative view is thought to be complementary to the one in the text.

The model is a two-dimensional tissue model representing the L1 layer of cells.

1.3.1. Quasi-static equilibrium (bio)mechanical module: mechanical stresses and strains

The first process is a purely mechanical process, the calculation of the mechanical stresses and strains required for the quasi-static mechanical equilibrium of the wall mesh under the load of the pressure of inner tissues. To do so, it is necessary to define the material element and its rheological behavior, the mechanical structure of the cell-walls, and the load.

Material scale: cell wall behavior: The material element under consideration is a piece of cell-wall. This wall is assumed to behave as a linear elastic material, so that when loaded it produced an elastic reaction through straining, and the resulting elastic stresses are proportional to its strain.

$$\sigma_w\left(n_w\right) = E_w \,.\, \epsilon_w^e = E_w \,.\left(\frac{l_w - l_{w0}}{l_{w0}}\right) \quad \text{Eq Sfm-(1)}$$

The coefficient of proportionality E_w is the stiffness of the wall material (its Young's modulus). As we may see later, only one dimensional stretching of the wall are considered. This wall elastic wall stiffness is under biological control as to be discussed later on

Structural scale: Geometrical modelling:

The previous wall elements are assembled into a two-dimensional tissue model representing a cell from the L1 layer of the SAM (and its connections with neighboring cells sharing side cell walls). The cell is represented as an hexagonal box. Each wall is of current length l_w and constant thickness t and depth d, and lays along its current direction n_w (with an angle θ_w to the long axis of the cell).

Note that only the lateral walls of the L1 cells were modelled. Each wall can be seen as a one dimensional spring (with elastic stiffness $k_w = E_w.t.d$) carrying a force F_w. The cell corners behave like ball-joints between the adjacent walls (no stiffness for changing angles)

Load and boundary condition: The load comes from the turgor pressure of the cells P, which is assumed to be homogeneous across all the meristem. Note that water flows into the expanding cells that are concurrently involved in growth are neglected here (see (Moulia and Fournier, 2009) for a review of this coupling). Then the consequences of this turgor pressure on the cell under concern can be split into two components: the action from the inner layers, and that from lateral cells

The load from the inner layers is modeled as a pressure from below the L1 cell, which is assumed to be equal to the turgor pressure of the cells of the inner layers (their walls are assumed to be so compliant that the whole pressure load is carried by the walls on the cells of the L1 layer). This inner pressure is thus putting the L1 layer into bi-dimensional tension and the underlying surface into a curved configuration so that local curvature allows for a balance between the inner force and the tensile reaction (i.e. wall stresses) within the L1 wall.

The lateral forces resulting from the turgor pressure of neighboring cells cancel themselves as turgor pressure is supposed to be homogeneous.

The boundary conditions are no displacement at the bottom of the SAM

Quasistatic force balance :

The force distribution is estimated from the force balance between the inner pressure and the (tensile) elastic reaction in the L1 cell walls.

The force from the internal tissue is given by

$$F_{c,int} = P_{c,int}.A_c \quad \text{Eq Sfm-(2)}$$

where A_c is the cell area and acts perpendicular to the two-dimensional cell plate

As stated earlier, the forces between cells of the L1 layer acting perpendicular to the wall are canceled due to the assumption of equal pressure in all cells.

The lateral walls of the cell hold (i.e. balance) the force $F_{c,int}$ due to the internal pressure by behaving as elastic elements with constant thickness (t) and depth (d), and linear elastic properties E_w, which results in a one-dimensional spring model for each wall , i.e. the outer and inner wall have been neglected in this model.

The force F_w along a given wall or direction n_w comes as

$$F_w\ (n_w) = k_w.\epsilon_w^e = k_w.\left(\frac{l_w - l_{w0}}{l_{w0}}\right) = E_w.t.d\left(\frac{l_w - l_{w0}}{l_{w0}}\right) \quad \text{Eq Sfm-(3)}$$

where F_w is the force acting along the one-dimensional spring direction n_w, k_w is the elastic stiffness of the wall k_w=t.d.E_w , l_w and l_{w0} are the actual and the rest (i.e. relaxed) wall lengths, respectively.

The elastic equilibrium configuration that is the amount of strains in the cell walls to achieve mechanical equilibrium between the load by inner pressure and the elastic reaction forces in the walls is then found by minimizing the elastic energy of the system

This module outputs the current wall-strain state to the growth module.

1.3.2. Meristematic growth (biomechanical) module

As the meristematic cells of the SAM are all undergoing cell division cycle, this means interphasic expansion, eventually followed by cell division.

The interphasic expansion is supposed to be a visco-plastic creep at a rate that is proportional to the actual elastic strain of the cell wall above a yield threshold (this model assumes that elastic and visco-plastic elements act in a serial form, and is equivalent to the Bingham flow rheology used in

Lockhart's model. But it is also consistent with the idea of some inclusion of new cell wall material into the wall, although the way that this is possible is not specified in the model).

Wall growth is obtained by increasing the resting length of walls at a rate that depends on the amount of elastic stretching of the wall above a yield-threshold following the dynamics

$$\frac{dl_w^0}{dt} = k_g \,.\left(\varepsilon_w^e - T_g\right) = k_g \,.\left(\frac{l_w - l_{w0}}{l_{w0}} - T_g\right) \qquad \text{Eq Sfm-(4)}$$

where k_g is the growth extensibility coefficient. Since k_g is constant, individual wall growth rates result from different elastic strains :

$$\varepsilon_w^e = \left(\frac{l_w - l_{w0}}{l_{w0}}\right)$$

Cell wall synthesis is (implicitly) assumed to be driven by the strain rate so to keep the depth d and thickness t of the wall constant (compensating for Poisson thinning effects during streching)

Cell division occurs when cells reach a threshold size, $A_{c\ max}$, and is implemented by adding a new wall through the center of mass of the cell, and in a direction parallel to the cortical microtubules direction of the cell Θ_c (to be defined more precisely in the next §). To avoid too short cell walls and 4-vertices, the connection of the new wall to old walls are moved to a minimal distance $0.3l_w$ from an existing vertex (along wall w).

1.3.3. Wall Stress Mechanosensing module

Mechanosensing is based on the hypothesis that the microtubule direction for a cell is sensitive to the direction of the mean principal stress in the cell. This response is not instantaneous but goes at a characteristic rate (which is independent from the stress state). . This can be calculated as the directional weighted average of the wall tension forces F_w using circular statistics with a periodicity of 180 deg, yielding:

$$\theta_c = \theta_{c\sum\vec{F}} = \frac{1}{2}\tan^{-1}\left(\frac{\sum_c F_w \sin(2\theta_w)}{\sum_c F_w \cos(2\theta_w)}\right) \qquad \text{Eq Sfm-(5)}$$

where Θ_c (Θ_w) is the directional angle for the cell (wall).

This is supposed to be the only mechanosensitive step in the model (for example no effect on auxin transport is implemented)

The second equation accounts for the fact that the changes in microtubule orientation cannot be

immediate, so that the CMTs direction is updated with a delay :

$$n_c^{new} = (1 - \alpha.\delta t).n_c^{old} + (\alpha.\delta t)n_c^{new} \qquad \text{Eq Sfm-(6)}$$

where n_c^{new} is the new direction, n_c^{old} is the previous direction, α is the time-rate for , δt is the time step.

This equation simply states that only a proportion $\alpha.\delta t$ of the total microtubule population has shifted to the new direction n_c specified by mechanosensing (independently of the stress)

1.3.4. Microtubule influence on elastic strain field

The longitudinal elastic stiffness of the wall E_w is supposed to under a biological control, modelled as depending on i) microtubules orientation in the same cells (θ_{c1} and θ_{c2}), ii) the concentrations of auxin in the adjoining cells on both side of the wall.

Microtubule influence on wall elastic stiffness (anisotropy)

Microtubule direction in each cell is supposed to act by driving changes in the mechanical anisotropy of the cell wall elastic stiffness by orienting the laying of cellulose microfibrils (along the same direction as the microtubules). E_w is thus supposed to depend on microtubules orientation in the same cells (θ_{c1} and θ_{c2}), according to:

$$E_w = E_{min} + E_{max}\left(\frac{\cos^2(\theta_1)+\cos^2(\theta_2)}{2}\right) \qquad \text{Eq Sfm-(7)}$$

E_{min} is the elastic stiffness of the isotropic CW matrix, and $E_{max}\left(\frac{\cos^2(\theta_1)+\cos^2(\theta_2)}{2}\right)$ is a stiffening term related to the direction angle Θ of the microtubules (and hence microfibrils) relative to the wall direction on both sides of the cell wall (this $\cos^2(\Theta)$ angular dependency is only specifying mathematically the idea that parallel and antiparallel orientations both lead to the maximal longitudinal stiffening, whereas perpendicular orientation leads to no stiffening).

The spring constant of the cell wall, k_w is then a function of the microtubules and wall direction

$$k_w = kE_w t.d = k_{min} + k_{max}\left(\frac{\cos^2(\theta_1)+\cos^2(\theta_2)}{2}\right) \qquad \text{Eq Sfm-(8)}$$

where k_w is the spring stiffness of a given wall, k_{min} is the isotropic contribution to k_w, k_{max} is the anisotropic contribution, n_c is the microtubule direction, n_w is the direction of the wall Θ is the angle between the cortical microtubule direction and the wall direction in the cell

Other influences on wall rheology

The elastic stiffness of the wall E_w (and hence k_w) is also assumed to be dependent on auxin

concentration on the two adjacent cell (Jonsson et al., 2006). This was implemented in the model through the an auxin-dependent factor

$$k_w\left(a_{c1}^{n_a}, a_{c2}^{n_a}\right) = k_w^0\left(\frac{K_a^{n_a}}{K_a^{n_a}+a_{c1}^{n_a}} + \frac{K_a^{n_a}}{K_a^{n_a}+a_{c2}^{n_a}}\right) \quad \text{Eq Sfm-(9)}$$

where $a_{c1}^{n_a}$ and $a_{c2}^{n_a}$ are the auxin concentrations in the neighboring cells and k_w^0 is the basic wall stiffness

This auxin- sub-model states that the elastic stiffness of the wall is decreased by auxin following an hyperbolic function ((high auxin concentration decreases wall stiffness, but this decrease is much faster for increment of auxin concentration when the initial concentration is low).

Note finally that in the application of the cell SFm to the whole SAM k_w^0 was supposed to depend on distance from the top of the dome.

1.3.5. Elastic strain feedback on growth

Due to the dependency of growth rate on the elastic strains of the wall (Eq Sfm-(4)), a decrease in wall elastic stiffness (for the same mechanical loading) will increase the expansion rate. Moreover, the size threshold for cell division will be reached sooner, also enhancing cell division rate. This will change the overall mechanical structure and thus the strain field, and do on.

2. Supplementary Figures

Supplementary Figure 1. The Wall-Stress feedback model by processes

The elastic stress and strain fields at time t under the load by the pressure of inner tissues is determined through quasi-static mechanical equilibrium (minimizing the total mechanical energy of the system). The cell-walls are assumed to behave as linear elastic bodies in tension, but their elastic Young's modulus E_w can differ from wall to wall. Cell corners are free to shear. This module outputs the current wall-strain state to the growth module.

Expansion growth is occurring proportionally to this elastic strain, above a yield threshold. The depth d and thickness t of the wall are assumed to be constant (wall deposition just balances Poisson's ratio effects to prevent the wall from thinning while being strained longitudinally). Growth-strains are the computed. Cell division occurs when the (rest) length of the cell overcomes a threshold, and is laid down in the mean direction of cortical microtubules (CMTs).

The Meristematic Growth module outputs the current rest lengths of all the walls (and their meshing

as a cellular structure)

In the mechanobiological module of the SFm, the mechanosensitive step occurs at the level of the cell. The central hypothesis of the module is that CMTs are re-aligned to the current direction of the cell-resultant direction of the stresses in the side walls θc, but this occurs at a constant pace with only a part of the overall CMTS population (n_{cnew}) being reoriented at each time step (this rate is assumed to be independent from the mechanical state). The current θc and n_{cnew} are then transmitted to the wall-rheology module.

In the wall-rheology module the current mean orientations of CMTs in the two cells jousting a given cell-wall θ1, Θ2 (which may not have reached the target orientations θc1, Θc2 due to the delay in CMTs reorientation) determine the longitudinal elastic stiffness of the side cell-wall E_w (presumably through the orientation of the laying of the new cellulose microfibrils with respect to the wall, changing the anisotropy of the cell wall elastic rigidity and hence its longitudinal stiffness, although the process is here assumed to be instantaneous). This latter process occurs at the level of each cell-wall element (so that elastic stiffening usually differ between the different walls of the same cell). When applying the SFm to the whole SAM, a positional dependency of E_w is implemented, as well as an hyperbolic dependency to the mean auxin content in the cell. The new value of wall elastic stiffness E_w is the output of the wall rheology module and is transferred to the quasi-static mechanical equilibrium module, in which the elastic stiffness is updated, changing immediately the constitutive law of the cell walls, and thus giving rise to a new mechanical equilibrium at the next time step.

3. References

Coutand, C., Chevolot, M., Lacointe, A., Rowe, N., and Scotti, I. (2010). Mechanosensing of stem bending and its interspecific variability in five neotropical rainforest species. *Annals of Botany* 105**,** 341-347. doi: Doi 10.1093/Aob/Mcp286.

Coutand, C., Martin, L., Leblanc-Fournier, N., Decourteix, M., Julien, J.L., and Moulia, B. (2009). Strain Mechanosensing Quantitatively Controls Diameter Growth and PtaZFP2 Gene Expression in Poplar. *Plant Physiology* 151**,** 223-232. doi: DOI 10.1104/pp.109.138164.

Coutand, C., and Moulia, B. (2000). Biomechanical study of the effect of a controlled bending on tomato stem elongation: local strain sensing and spatial integration of the signal. *Journal of Experimental Botany* 51**,** 1825-1842. doi: DOI 10.1093/jexbot/51.352.1825.

Jonsson, H., Heisler, M.G., Shapiro, B.E., Meyerowitz, E.M., and Mjolsness, E. (2006). An auxin-driven polarized transport model for phyllotaxis. *Proceedings of the National Academy of Sciences of the United States of America* 103**,** 1633-1638. doi: DOI 10.1073/pnas.0509839103.

Moulia, B., Coutand, C., and Lenne, C. (2006). Posture control and skeletal mechanical acclimation in terrestrial plants: Implications for mechanical modeling of plant architecture. *American Journal of Botany* 93**,** 1477-1489. doi: DOI 10.3732/ajb.93.10.1477.

Moulia, B., Der Loughian, C., Bastien, R., Martin, L., Rodriguez, M., Gourcilleau, D., Barbacci, A., Badel, E., Franchel, J., Lenne, C., Roeckel-Drevet, P., Allain, J.M., Frachisse, J.M., De Langre, E., Coutand, C., Fournier-Leblanc, N., and Julien, J.L. (2011). "Integrative mechanobiology of growth and architectural development in changing mechanical environments," in

Mechanical Integrative of Plant Cells and Plants, ed. P.W. (Ed). (Berlin (Allemagne): Springer-Verlag), 269-302.

Moulia, B., and Fournier, M. (2009). The power and control of gravitropic movements in plants: a biomechanical and systems biology view. *Journal of Experimental Botany* 60**,** 461-486. doi: Doi 10.1093/Jxb/Ern341.

Schriefer, J.L., Warden, S.J., Saxon, L.K., Robling, A.G., and Turner, C.H. (2005). Cellular accommodation and the response of bone to mechanical loading. *Journal of Biomechanics* 38**,** 1838-1845. doi: DOI 10.1016/j.jbiomech.2004.08.017.
