# Supplementary figures and images for: Mechanosensitive control of plant growth: bearing the load, sensing, transducing, and responding
Source: Front Plant Sci. 2015 Feb 23;6:52. doi: 10.3389/fpls.2015.00052 (PMC4337334; doi:10.3389/fpls.2015.00052)

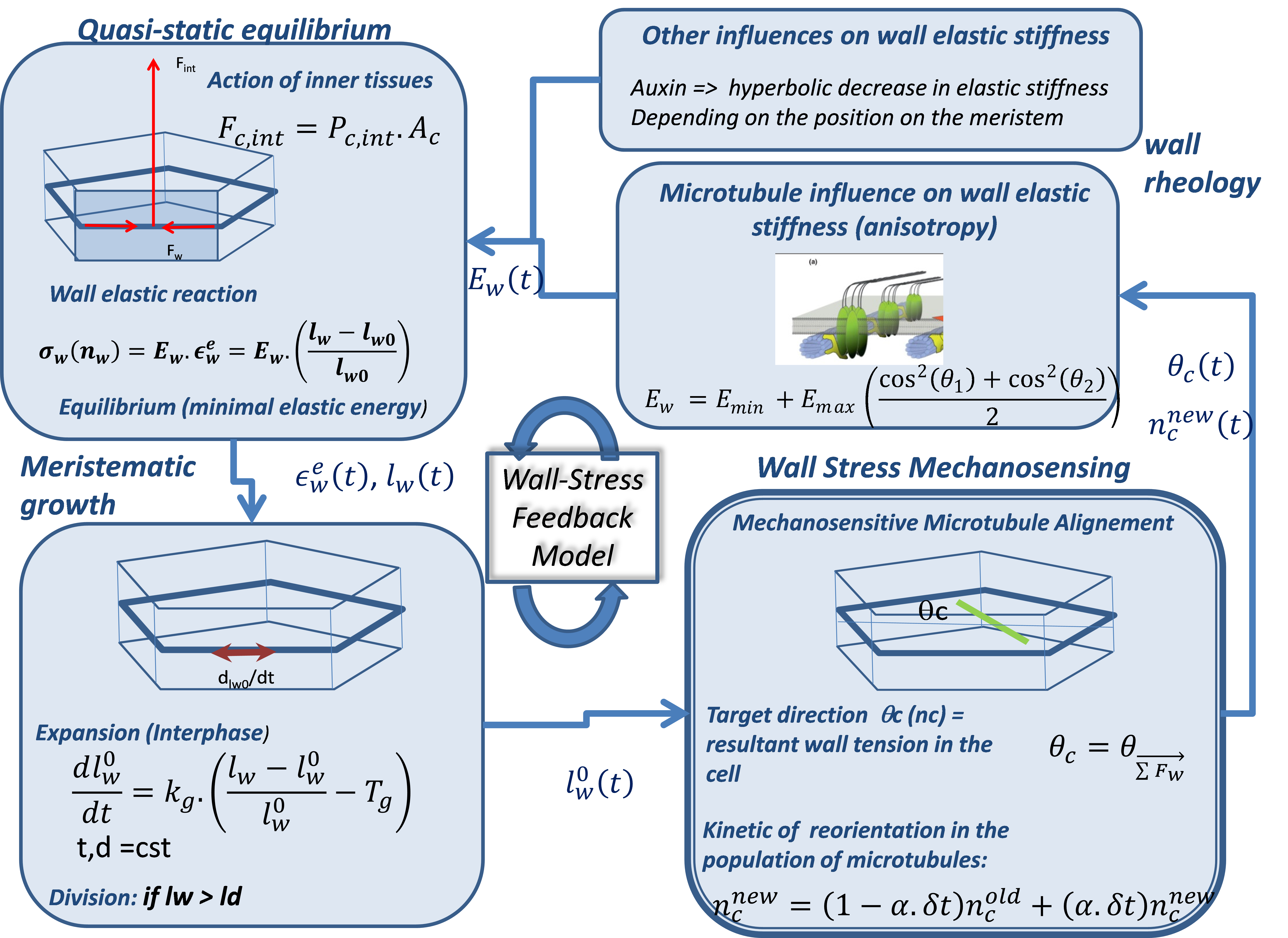

Supplement: Supplementary file 2 [file Image1.TIF]
